# An actin mechanostat ensures hyphal tip sharpness in *Phytophthora infestans* to achieve host penetration

**DOI:** 10.1126/sciadv.abo0875

**Published:** 2022-06-10

**Authors:** Jochem Bronkhorst, Kiki Kots, Djanick de Jong, Michiel Kasteel, Thomas van Boxmeer, Tanweer Joemmanbaks, Francine Govers, Jasper van der Gucht, Tijs Ketelaar, Joris Sprakel

**Affiliations:** 1 Physical Chemistry and Soft Matter, Wageningen University & Research, Stippeneng 4, 6708 WE Wageningen, Netherlands.; 2 Laboratory of Phytopathology, Wageningen University & Research, Droevendaalsesteeg 1, 6708 PB Wageningen, Netherlands.; 3 Laboratory of Cell Biology, Wageningen University & Research, Droevendaalsesteeg 1, 6708 PB Wageningen, Netherlands.; 4 Laboratory of Biochemistry, Wageningen University & Research, Stippeneng 4, 6708 WE Wageningen, Netherlands.

## Abstract

Filamentous plant pathogens apply mechanical forces to pierce their hosts surface and penetrate its tissues. Devastating *Phytophthora* pathogens harness a specialized form of invasive tip growth to slice through the plant surface, wielding their hypha as a microscopic knife. Slicing requires a sharp hyphal tip that is not blunted at the site of the mechanical interaction. How tip shape is controlled, however, is unknown. We uncover an actin-based mechanostat in *Phytophthora infestans* that controls tip sharpness during penetration. Mechanical stimulation of the hypha leads to the emergence of an aster-like actin configuration, which shows fast, local, and quantitative feedback to the local stress. We evidence that this functions as an adaptive mechanical scaffold that sharpens the invasive weapon and prevents it from blunting. The hyphal tip mechanostat enables the efficient conversion of turgor into localized invasive pressures that are required to achieve host penetration.

## Teaser

A cytoskeletal mechanostat is discovered in a plant pathogen that allows it to sharpen its knives for host penetration.

## INTRODUCTION

Host entry is the first step in the disease cycle of biotrophic filamentous plant pathogens (*1*–*4*). To achieve penetration, pathogens adhere to the host surface (*5*, *6*) and apply substantial invasive forces (*7*–*10*) to induce a mechanical fracture in the plant cuticle and epidermis that serves as a port of entry. Although the two main phylogenetic groups that encompass filamentous plant pathogens, fungi and oomycetes, diverged between 1.2 and 1.7 billion years ago (*11*), it is commonly held that they developed similar strategies for host penetration (*4*, *12*, *13*). Nonetheless, marked differences between fungi and oomycetes are apparent, although host penetration mechanics remain relatively poorly studied for pathogens in the group of oomycetes.

Plant pathogens harness a variety of mechanisms to gain first entry into host tissues from the exterior. Many fungi enter plants via natural openings, such as stomata or wounds, while others use direct penetration by invasive force generation to pierce through the plant cuticle and epidermis (*14*). Direct penetration by fungi can, for example, occur from germ tubes or mycelium by outgrowths that penetrate epidermal cells and develop into haustoria or grow further intercellular or from germinating spores that form a specialized invasion structure known as an appressorium (*15*), which can build tremendous turgor pressures (*7*, *9*). Appressorial penetration through the mechanical barrier of the plant surface involves converting the stored mechanical energy in the highly turgescent cell into penetrative forces by ejecting penetration peg that pierces the plant surface. Appressorium formation, following a recent definition (*15*), involves a change in cellular identity, a transition from polarized hyphal growth to nonpolar growth. Appressorial penetration is often mediated by septins (*16*, *17*), guanosine triphosphate (GTP)–binding cytoskeletal proteins that form filaments, and can involve the formation of a melanized shell around the cell that acts as a pressure-containment vessel, for example, in the well-studied rice blast fungus (*18*, *19*). To mediate direct penetration, fungi also use biochemical weapons, e.g., by secreting cutinases and/or cell wall–degrading enzymes, which can weaken the mechanical barriers (*14*).

Oomycete host penetration strategies share similarities to those used by their fungal counterparts. Oomycete host entry also involves the conversion of turgor to penetrative forces, and lytic enzymes are secreted from their hypha, such as those that target pectins, to weaken the cohesion of plant cell walls (*14*, *20*). However, marked differences are also evident. Oomycetes lack a melanin biosynthesis machinery and do not have septins; neither is invasion associated with a change in cellular identity nor a transition from polar to nonpolar growth. We recently uncovered the mechanical mechanism by which *Phytophthora* species, the most notorious and damaging oomycete plant pathogens, create a break in the plant surface, which to our current understanding differs fundamentally from what is known for fungi (*8*). Once germinated, single-celled cysts of *Phytophthora* spp. achieve host entry by using their hypha as the invasive weapon. The hyphal tip focuses turgor to the contact point to generate localized invasive stresses. A distinct oblique angle of force application gives rise to strong stress localization near the surface and the emergence of tensile stresses, the combination of which greatly reduces the pressures required to induce a surface fracture, thereby providing the pathogen a point of entry. The mechanics of this mode of entry are analogous to the way a single-bevel Japanese kitchen knife facilitates the formation of a clean cut; hence, we termed this host entry strategy naifu invasion (*8*), after the Japanese word for knife.

Host entry by *Phytophthora* spp. is sometimes accompanied by hyphal swelling at the point of entry; these inflated germ tube sections are often referred to as either appressoria or appressorium-like structures (*21*–*24*). Because these structures lack several defining features of true appressoria (*15*), we argued previously that this terminology in the context of oomycete pathogenicity may be misleading (*8*). We hypothesize that these hyphal swellings, which are most often seen after surface penetration, are likely a collateral effect. Germ tube inflation under large turgor combined with the squeezing forces acting on the hypha at the site of entry may cause the tube to bulge, in the same way that inflating a balloon while squeezing a section of it will lead to a bulge near the constriction.

In essence, naifu invasion is a specialized form of invasive tip growth, a phenomenon studied extensively in plant pollen tube growth (*25*–*27*). Tip growth, during which a walled cell extends by expansion of the cell wall at its tip, is driven by converting isotropic turgor pressure into polarized expansion by modulating the local stiffness and the yield stress of the wall (*27*–*30*). Thus, tip growth involves a relative softening of the wall at its apex to achieve local viscoplastic flow (*29*) as the mechanism to direct polarized growth. Because application of indentation forces during host entry results from tip elongation (*8*), this same mechanism is most likely at the heart of invasive force application in oomycetes. In *Phytophthora*, this process is thought to be orchestrated by the actin cytoskeleton that has a polar architecture in the direction of growth and is believed to guide the transport of exocytotic vesicles to the site of wall expansion (*31*, *32*).

Without a scaffold to preserve tip shape, contact mechanics dictate that pressing a hypha into a surface of equal or larger stiffness under large indentation forces would invariably lead to tip flattening (*33*). In the same way that one cannot create cuts with a blunt knife, naifu invasion also requires a sharp hyphal tip to localize stresses to the intended entry point and prevent the loss of mechanical energy to the deformation of the pathogen itself. This creates a mechanical conflict in invasive growth via naifu invasion. Without additional mechanisms, such as the one we uncover here, a locally softened hyphal tip, as required for growth and force generation (*27*–*30*), would deform and flatten strongly upon mechanical contact with the cuticle that forms the outer protective layer of the host. In other words, these pathogens must simultaneously maintain tip softness to enable the conversion of isotropic turgor to directional growth and tip sharpness and stiffness to enable penetration. How this conflict is resolved remains elusive.

Here, we uncover an actin-based mechanostat in the potato late blight pathogen *Phytophthora infestans* that resolves this mechanical conflict. We find that mechanical stimulation of the hypha activates pronounced actin assembly and accumulation in vicinity of the site of mechanical contact. We combined live-cell cytoskeletal imaging with local force measurements to evidence fast, local, and quantitative feedback between the actin remodeling and the amplitude of the mechanical stress. We postulate, supported by mechanical modeling, that the force-gated actin remodeling results in an adaptive mechanical scaffold, whose stiffness is matched to the level of stress, that prevents tip deformations during host entry. Last, we show that mechanical stimuli lead to tip sharpening and the maintenance of tip sharpness even under large blunting stresses. This feedback mechanism constitutes a tip shape mechanostat that enables hyphal tips to remain malleable and sharp, thereby enabling invasive growth combined with efficient force localization to achieve host penetration.

## RESULTS

### Actin cytoskeleton remodels during invasive growth

**Fig. 1.**
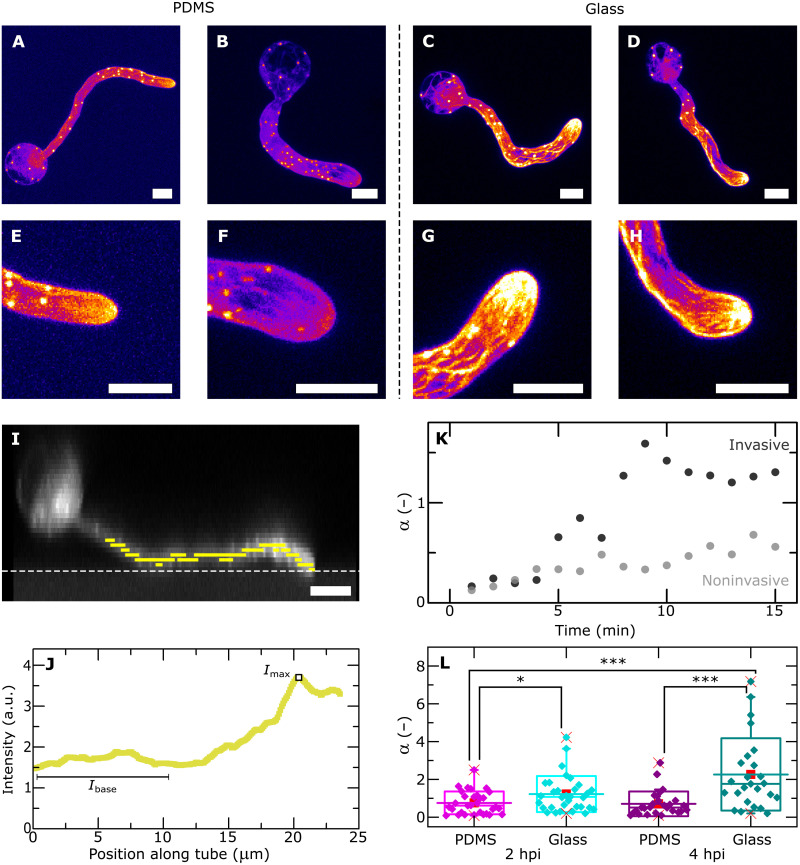
Substrate stiffness controls actin organization in invasive *P. infestans*. (**A** to **D**) Maximum intensity *xy* projections of hyphae of LifeAct-eGFP–expressing *P. infestans* (Pi-LA-GFP) on PDMS (A and B; *N* = 32 cells/3 independent experiments) and glass (C and D; *N* = 45 cells/3 independent experiments), with corresponding close-up images of the hyphal tips shown in (**E**) to (**H**). (**I**) *xz* projection of an invasively growing hypha on glass. Gray dotted line: substrate plane, yellow markers: germ tube centroid from image analysis, with the corresponding LifeAct-eGFP intensity as a function of position along the hypha in (**J**) (see fig. S4 for additional examples). a.u., arbitrary units. (**K**) Extent of actin accumulation in the hyphal tip expressed by parameter α (as defined in text) as a function of time for a single cell undergoing noninvasive growth (gray) versus a single cell that grows invasively into a PDMS substrate (black). *N* = 10 cells/6 independent experiments. (**L**) Box plot showing the distribution of α values for invasively growing hyphae on PDMS and glass substrates, 2 and 4 hours post-inoculation (hpi); a two-sided Wilcoxon rank sum test was used to compare α between glass and PDMS at 2 and 4 hpi, respectively (*a* < 0.05, *P > 0.02, ***P < 0.01). *N* = 31 cells/4 independent experiments (PDMS—2 hpi), *N* = 27 cells/2 independent experiments (PDMS—4 hpi), *N* = 32 cells/3 independent experiments (glass—2 hpi), and *N* = 27 cells/3 independent experiments (glass—4 hpi). Scale bars, 5 μm (A to I).

The actin cytoskeleton is well known to play a crucial role in the polarized tip growth of hypha in *P. infestans* (*31*, *32*). We visualized the actin cytoskeleton by live-cell imaging of a transgenic *P. infestans* line, abbreviated Pi-LA-GFP (*32*), expressing the F-actin binding LifeAct–enhanced green fluorescent protein (eGFP) marker (*34*). In line with previous reports (*31*, *32*), we observe several distinct cytoskeletal configurations. Germinated cysts show bright diffraction-limited spherical entities (Fig. 1, A to H, and fig. S1), known as plaques, whose precise function and internal structure remain elusive, but which are thought to play a role in actin organization and correlate to hyphal morphology (*32*, *35*–*37*). Second, F-actin occurs as a network of interconnected cables of varying thickness that occupies the cytosol of the germ tube, but whose intensity is highest at the hyphal tip (Fig. 1, A to H, and fig. S1). This tip-focused actin network is thought to play an important role in regulating tip growth by directing the transport of exocytotic vesicles filled with new cell wall material to the growing tip (*31*, *32*).

We asked the question whether the actin cytoskeleton, in addition to playing a role in the regulation of polarized growth, could play a mechanical role as well. Previous studies showed that contact with a glass substrate triggers a remodeling of the tip-localized network to an aster-like structure with thick actin cables emanating from the contact point with the surface and extending many micrometers into the germ tube posterior (*31*). We visualized the actin cytoskeleton during interaction of the tip with two substrates whose stiffness differs by many orders of magnitude: glass, with a stiffness expressed by the Young’s modulus of *E* ~ 10^11^ Pa, and a soft silicone rubber [polydimethylsiloxane (PDMS)] with *E* = 0.6 × 10^6^ Pa (*8*). Here, we define noninvasive growth as germ tube extension without mechanical interactions with the substrate, and invasive growth as the phase during which the germ tube growth is slowed substantially because of mechanical forces emerging between hypha and substrate. We find that both substrates induced a transition from noninvasive to invasive growth, which coincides with a remodeling of the actin cytoskeleton from a finely meshed network into a tip-localized structure consisting of F-actin cables in an aster-like geometry (Fig. 1). These effects are substantially more pronounced on the stiff substrate (Fig. 1, C, D, G, and H) than on the softer surface (Fig. 1, A, B, E, and F). These data provide a first hint to the force-gated remodeling of the cytoskeleton involved in invasive growth. We confirmed that the increased fluorescence and alterations in microstructure observed in the tip of the Pi-LA-GFP actin marker line are not simply due to an increased accumulation of cytoplasm in the apical region of the germ tube. Control experiments in a *P. infestans* line expressing an unbound cytosolic GFP [Pi-14-3-GFP (*38*)] show virtually no fluorescence increase in the tip before substrate penetration and the lack of any microstructure (see the Supplementary Materials and fig. S2).

We note that previous studies on actin in hyphal tips of *Phytophthora* spp. during invasive growth report inconsistent localizations. A study that stained the actin cytoskeleton with fluorescent conjugates of the actin-binding toxin phalloidin concluded that the hyphal tip is depleted of F-actin (*39*). By contrast, a study using LifeAct-eGFP as the actin reporter showed the same actin aster-like structure as we observe here and concluded that F-actin accumulates in the tip (*31*). To resolve this apparent contradiction, we compare hyphal tip images, growing invasively on PDMS substrates, stained by a phalloidin-rhodamine conjugate after fixation versus a live-cell imaging approach using LifeAct-eGFP (fig. S3). Only the LifeAct-eGFP images reveal the actin aster in the tip, while images of the fixed and phalloidin-stained *wt* strain show a depleted zone (see the Supplementary Materials). This could suggest that the previously reported F-actin depletion in the tip (*39*) is an artifact, either due to a lack of phalloidin binding to this structure or due to the fixation that precedes staining.

We quantified the extent of actin accumulation near the hyphal tip by means of an image analysis routine that first identifies the centroid contour of the germ tube from two-dimensional projections in the *xz* plane of three-dimensional image stacks obtained with confocal microscopy (Fig. 1I and fig. S4). We verified (fig. S5) that the orientation of the projection does not substantially influence the outcome of this analysis. Subsequently, we used the contour to measure the fluorescence intensity as a function of position in the germ tube (Fig. 1J). In virtually all germ tubes we analyzed, we observed a distinct increase in F-actin accumulation near the tip. We defined the excess of F-actin in the hyphal tip as $\alpha =\frac{I_{\text{max}}}{I_{\text{base}}}-1$, where *I*_max_ is the peak intensity, located at or near the tip, and *I*_base_ is the mean baseline intensity, recorded in the posterior section of the germ tube (Fig. 1J). If the F-actin concentration is homogeneous along the germ tube, α = 0, while strong tip accumulation gives α >> 0.

We first observed that actin accumulation increases during germ tube initiation and growth also in noninvasive hyphae that do not experience measurable mechanical contact with the substrate (gray symbols in Fig. 1K), with α reaching a maximum value of ~0.5. This reflects the polar actin structure involved in regulating tip growth in the absence of mechanical interactions. By contrast, in hyphae that push into a substrate and experience compressive mechanical stresses at their tip, the actin accumulation is more pronounced, with α increasing to well above 1 (black symbols, Fig. 1K). By aggregating data for multiple cells, we find that actin accumulation at the tip is significantly enhanced on stiffer substrates as compared to soft surfaces, especially during later stages of the growth process (Fig. 1L). Mechanical interactions with a substrate influence the extent to which the cytoskeleton remodels and accumulates near the hyphal tip.

**Fig. 2.**
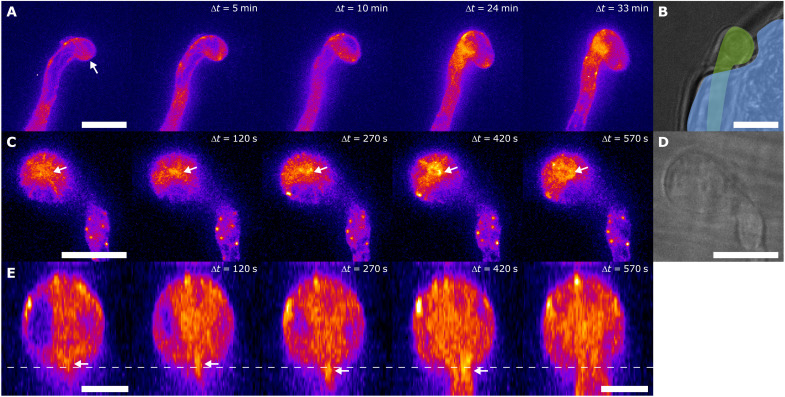
Mechanically-induced actin remodeling during pathogenic cell penetration. (**A**) Time sequence of confocal microscopy images of *P. infestans* Pi-LA-GFP during attempted penetration of a suspension-cultured tomato MsK8 cell, showing actin aster formation (indicated with arrow) at the site of contact (*N* = 4 cells/1 independent experiment). (**B**) Bright-field fluorescence overlay image shows the pathogen (green) indenting the dead tomato cell (blue) that has lost turgor pressure. Scale bars, 10 μm (A and B). (**C**) Singular unique observation of Pi-LA-GFP during penetration of an etiolated potato stem. Penetration occurs between 270 and 300 s. (**D**) Bright-field image of invasive hypha shown in (C). (**E**) *xz* projections showing an orthogonal view (90° rotation) of the images in (C). Scale bars, 10 μm (A to D) and 5 μm (E). Arrows in (A), (C), and (E) indicate aster, and the dashed line in (E) indicates the stem surface.

A similar response of the actin cytoskeleton was observed in several unique observations made during attempts of *P. infestans* to enter plant cells. During the host entry attempt of Pi-LA-GFP on suspension-cultured cells of the tomato MsK8 cell line (*40*), we made a very low number of observations (*N* = 4) on hypha that attempted to penetrate a dead cell (Fig. 2, A and B). The penetration attempt caused the cell wall of the tomato cell to deform inward, but penetration was unsuccessful (Fig. 2B), potentially indicating that a host cell needs to be turgescent for successful penetration. Nonetheless, in our few observations, an actin aster was clearly visible at the site of mechanical contact (arrow in Fig. 2A). In addition, we serendipitously imaged a singular penetration event on in vitro grown potato plantlets (Fig. 2, C to E); we observe an actin aster (marked with an arrow in Fig. 2, C and E), the center of which localizes to the indentation location. In this observation, we find penetration to occur at a time 270 to 300 s after starting the imaging (see also fig. S7A). We find that the actin fluorescence peaks approximately 1 min after penetration, after which the aster gradually disappears (Fig. 2C and fig. S7B). These few observations hint at the fact that the timing and localization of the actin aster correlate with the formation of mechanical interaction with a substrate or cell, and that the relaxation of the mechanical stress, by rupture of the surface, leads to disappearance of the actin aster. This suggests that the actin aster is only present when a mechanical force is active. These observations triggered us to study these processes more rigorously.

### Cytoskeletal remodeling upon laser ablation

The mechanically induced actin restructuring described above appears to occur almost instantaneously when a mechanical stimulus emerges at the hyphal tip by contact with either an artificial surface or a plant cell. Because of the limited time resolution in these experiments, it is difficult to quantify the kinetics of the remodeling. Since invasion occurs by indenting a substrate with the hyphal tip, we only observe the aster formation there; whether this is a specific feature of the cytoskeleton in the tip or an intrinsic feature of the entire cytoskeleton thus remains unanswered.

Laser ablation has been extensively used in other systems to induce a local disturbance in cytoskeletal networks by creating photothermal damage inside the cell as the result of strong local laser irradiance and to explore how the cytoskeletal networks respond to this acute upset of the mechanical balance of the biopolymer network (*41*, *42*). Photothermal damage to the proteins in the ablation zone, here targeted to either actin cables or plaques, which are visible with the LifeAct-eGFP F-actin marker, causes a strong and acute disturbance in the mechanical balance of the cytoskeleton. We note that this approach to create a mechanical stimulus is not directly comparable to the mechanical stimulation of the hyphal tip at a substrate-tip contact zone; laser ablation results in an acute damage to the cytoskeleton, and other structures in the ablation zone, while the mechanics at the hyphal tip during penetration are more moderate and sustained. Nonetheless, it offers a way to create a local and acute disturbance of the cytoskeletal mechanical balance (*41*, *42*), to study how the network responds to such a stimulus.

**Fig. 3.**
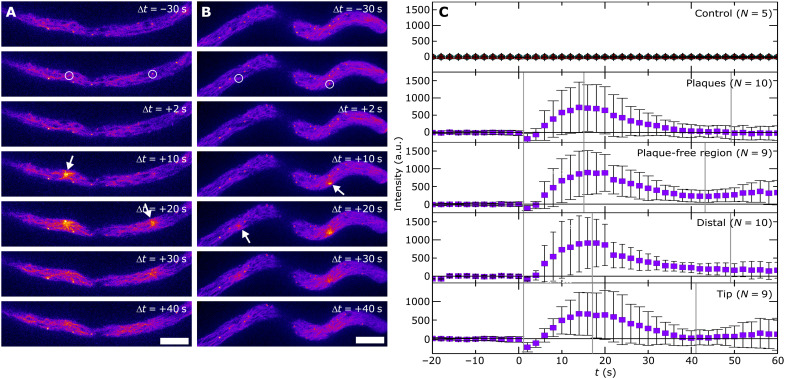
Kinetics of cytoskeletal remodeling upon local stimulation. Live-cell imaging of *P. infestans* Pi-LA-GFP upon laser ablation at *t* = 0 in a distal area of the hypha (**A**) and in proximity of the hyphal tip (**B**) during a time frame of 40 s. In each hypha, the ablation laser was targeted to a plaque and to a plaque-free region of the cytoplasm (indicated with circles), respectively. Actin asters are indicated by arrows. Scale bars, 5 μm (A and B). (**C**) Fluorescence intensity in the ablation zone as a function of time, with *t* = 0 defining the time of the ablation pulse, for a cytosolic eGFP line (control, Pi-14-3-GFP) and the actin marker line (Pi-LA-GFP) for two different targets (plaques and plaque-free regions) and two different locations in the hypha. All curves are averaged over several repeated measurements (as indicated); symbols denote average normalized intensities, and error bars denote the SD across the repeated experiments (number of repeats as indicated). Error bars in the control experiment are smaller than the symbol size. Vertical gray lines indicate the start of the formation and dissolution phase, respectively.

We pointed an ablation laser to both the hyphal tip region and to a region distal from the tip and selected actin plaques or plaque-free regions as targets. Notably, independent of the position of ablation and the target, laser ablation leads to the rapid formation of cables organized in an aster-like geometry (Fig. 3, A and B, and fig. S6). In all cases, the center of the aster is located at the laser ablation spot. We also observe that for tens of seconds after application of the stimulus, the aster spontaneously disappears.

To quantify the kinetics of this process, we recorded the temporal changes in LifeAct eGFP intensity in the ablated zone, with 2-s resolution, and normalized this to the baseline intensity in that zone before ablation, defining *t* = 0 as the moment of stimulation. We aggregated data from multiple experiments for the same location or the same target (Fig. 3C). For all locations and ablation targets, we observe a rapid increase in LifeAct fluorescence, which signals the formation of the actin aster. The formation process is completed in 15 ± 1 s after ablation. Once the stimulus is removed, the cytoskeleton relaxes to its unperturbed configuration. This spontaneous disassembly process is completed in 29 ± 5 s. We note that these effects are absent in control experiments on a *P. infestans* line that expresses a cytosolic eGFP marker (PI-14-3-GFP; Fig. 3C), indicating that our observation relates to cytoskeletal reorganization and not to a disturbance of the entire cytosol.

These data provide several important clues about the mechanically induced changes in cytoskeletal structure. First, we observe that the formation of the actin aster is not specific to the hyphal tip but appears to be an intrinsic feature of the cytoskeleton throughout the hypha. Second, we find that the formation of the actin aster in response to photothermal damage is very local and rapid, occurring at the ablated site and requiring only 15 s to complete. Last, this structure appears to be under mechanical control: It only forms when a stimulus is applied and vanishes again within half a minute after the stimulus is removed.

### Quantifying mechanical feedback

**Fig. 4.**
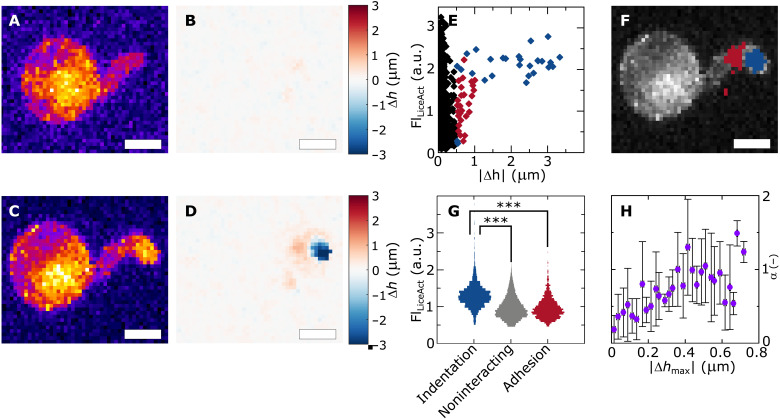
Local and quantitative mechanical feedback to cytoskeletal organization. (**A** to **D**) LifeAct-eGFP imaging combined with local surface mechanics measurements, showing *xy* projections of *P. infestans* Pi-LA-GFP during invasive growth on a PDMS substrate (A and C) and corresponding surface deformation maps (B and D) in the phase just before (A and B) and during force generation (C and D). (**E**) Scatterplot, showing the local LifeAct eGFP intensity (*y* axis) and absolute surface deformation amplitude for each pixel at a single time point for a single cell (*x* axis); data are grouped into adhesive (red), indentation (blue), and noninteracting sites (black) on the basis of the sign and amplitude of the local surface deformation (as explained in text). (**F**) Overlay of identified adhesion (red) and indentation sites (blue) on the *xy* projection of the LifeAct eGFP signal for a single cell showing strong localization in two distinct zones; additional examples shown in fig. S8. Spatial correlation of LifeAct-eGFP and surface deformations was performed on *N* = 6 cells/5 independent experiments. (**G**) Violin plot showing the distribution of LifeAct eGFP intensities associated with the three distinct sites, collected from *N* = 6 cells and *n* = 5 time points per cell, resulting in a total number of data points of 26,000 (indentation), 232,000 (noninteracting), and 25,000 (adhesion). A two-sided unpaired Student’s *t* test with Welch’s correction was performed to compare relative fluorescence intensities in the indentation zone versus the noninteracting and adhesion regions (*a* < 0.05 and ***P < 0.01). (**H**) Quantitative correlation between the extent of actin accumulation in the tip, expressed by parameter α (*y* axis), and the maximum amplitude of surface deformations (*x* axis) as a proxy for the applied invasive force. Diamonds represent means, and scale bars represent SDs between aggregated time series for *N* = 6 cells/5 independent experiments. Scale bars, 5 μm (A to D and F).

To quantify the extent to which mechanical forces elicit a structural remodeling of the actin cytoskeleton in the hyphal tip, we grow Pi-LA-GFP on surfaces that enable quantitative mapping of the surface forces involved in invasive growth. We use a previously developed approach that uses fluorescent elastomer surfaces and image analysis to measure the axial deformation of the substrate Δ*h* induced by the pathogen (*8*). Invasion on these surfaces begins with hyphal extension without mechanical contact, thereby leaving the substrate surface undeformed (Fig. 4, A and B). After a switch to invasive growth, the pathogen adheres to the surface and applies downward invasive force onto the surface to induce a fracture to gain entry (Fig. 4, C and D). This results in a distinct asymmetrical deformation pattern of an adhesion site (upward deformations, red in Fig. 4D) and an indentation zone (downward deformations, blue in Fig. 4D). The mechanics of these phases was discussed in more detail previously (*8*).

This approach allows us to simultaneously measure, in each pixel, the LifeAct-eGFP intensity, albeit at a lower resolution (Fig. 4, A and C) than for the experiments presented in Fig. 1, and the amplitude of surface deformations Δ*h* (Fig. 4, B and D). We begin by asking our data whether the mechanically induced actin remodeling is a local phenomenon as suggested by our data presented above. We separate the data into pixels in adhesion sites (Δ*h* > +0.5 μm, blue), indentation sites (Δ*h* < −0.5 μm, red), and noninteracting sites (−0.5 < Δ*h* < +0.5 μm, gray). When we plot, for a single cell at a single time point, the actin intensity in each pixel as a function of the absolute amplitude of the surface deformation |Δ*h|,* we see how these three subpopulations occupy different spaces (Fig. 4E and fig. S8). Indentation sites, where stresses on the germ tube are compressive and which are localized to the tip (blue in Fig. 4F), correspond to high GFP fluorescence reflecting high local concentrations of F-actin, whereas adhesion sites, found posterior to the indentation site (red in Fig. 4F) and where stresses are tensile, show lower fluorescence pointing to lower actin concentrations. Zones, in which surface deformations are small or absent, on average correspond to low actin concentrations, except for the cyst body. The latter is responsible for the data points at low deformations and high intensities.

To quantify these observations further, we accumulated data for *N* = 6 cells and *n* = 5 time points per cell in a field of view surrounding the site of invasion, resulting in a total of 283,000 individual data points. Distributions of these data reveal a statistically significant increase in the F-actin concentration in pixels associated with indentation where the hypha experiences compressive stresses (red in Fig. 4G) as compared to noninteracting sites (gray in Fig. 4G). By contrast, in the adhesion sites, where the germ tube is also under mechanical stress of equal magnitude, due to force balance (*8*), but of opposing sign (predominantly tensile), we find no significant local increase in the F-actin concentration. The absence of a cytoskeletal response in the adhesion site suggests that the mechanical activation of the actin remodeling could be sensitive to the vectorial nature of the mechanical stimulus.

Using the same approach, we can also evaluate how the extent of actin accumulation in the tip, using the metric α, is sensitive to the amplitude of the mechanical stress. We measured the maximum indentation deformation |Δ*h*_max_|, which is proportional to the applied invasive force, as a function of time in 2-min intervals, aggregated for *N* = 6 cells during their invasive growth, and simultaneously determine α. We find a quantitative correlation between the amplitude of the surface deformation, as a measure for the applied indentation force, and the extent of actin accumulation at the hyphal tip, with α showing a near-linear growth with |Δ*h*_max_| (Fig. 4H). An increase in the applied force results in the instantaneous, within the time resolution of our measurements, increase of the extent of actin accumulation. These results show that the mechanical feedback exhibited by the actin cytoskeleton is not only local and rapid but also quantitative.

### Mechanostat hypothesis and model

Our results above demonstrate that the organization of the actin cytoskeleton in the hyphal tip of *P. infestans* is under mechanical control. It is likely that the observed mechanical feedback to the actin network plays a role in the mechanics of invasion. It is often presumed that actin plays little to no significant mechanical role in the invasive growth of walled cells, in part, because the cell wall rigidity and turgor are orders of magnitude larger than the stiffness of actin structures (*43*, *44*). Actin polymerization cannot produce sufficient force to deform the cell wall or generate invasive forces (*43*), and invasive forces are caused solely by directing turgor to a localized area. However, this does not exclude that, in addition to being an orchestrator of polar transport, the actin network can play a role as a mechanical scaffold to shape the tip. The other known structural protein filaments in *Phytophthora*, microtubules, do not form scaffold structures in the hyphal tip of (*45*, *46*) and are nonobligatory for hyphal tip morphogenesis and polarized growth (*47*). It thus seems likely that microtubules are not implicated in the responses we observe.

We hypothesize that the mechanically controlled actin structure in the hyphal tip serves as an adaptive mechanical scaffold, whose rigidity is matched by the quantitative feedback mechanism to the local and instantaneous level of mechanical stress, which maintains tip sharpness during invasion. This proposed mechanism thus constitutes an actin mechanostat: The cytoskeletal stiffness is adaptively tuned to the level of stress acting on the tip to ensure a constant tip shape during invasion to accommodate the increase of the mechanical stresses during invasion. If true, this mechanostat would allow the cell wall to become locally weakened at the tip to accommodate tip growth without sacrificing penetration efficiency, thereby resolving the mechanical conflict involved in naifu invasion.

Typical elastic moduli for actin networks range from tens to hundreds of kilopascals, depending on their architecture, degree of bundling, density, and cross-linking state (*48*–*52*), whereas cell walls can be as stiff as hundreds of megapascals (*53*–*55*), but estimated to be substantially weaker, at tens of megapascals, in growing tips (*26*, *56*). This large stiffness asymmetry raises the question whether the actin cytoskeleton is not too weak to play the suggested role of a tip shape scaffold. First, we realized that while stiff, the cell wall is a thin quasi–two-dimensional sheet, whereas the actin structure we studied above is not exclusively localized to the cell cortex; from our imaging, it appears to fill the three-dimensional space of the germ tube at its apex, thus having dimensions of several micrometers.

**Fig. 5.**
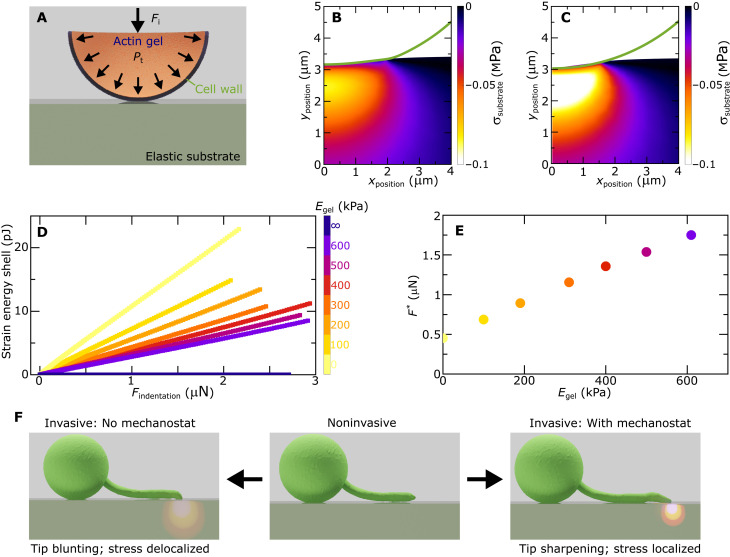
Mechanical model for cytoskeletal cell wall fortification. (**A**) Schematic illustration of the finite-element mechanical model. (**B** and **C**) Shape of the cell wall (green line) and stress distribution in the substrate for a tip without actin gel (B) and a tip with an infinitely stiff actin gel (C). The total indentation force is 1 μN in both cases. The color scale in the substrates indicates the principal compressive stress as the result of tip indentation. (**D**) Total strain energy in the cell wall (*y* axis), as a measure for the extent of tip deformation, as a function of the indentation force (*x* axis) for tips containing actin gels of different modulus; color scale expresses the actin gel modulus. (**E**) Indentation force F^*^ (*y* axis) at which the strain energy in the shell reaches a threshold value of 5 pJ as a function of the modulus of the actin gel (*x* axis). (**F**) Schematic illustration of hyphal tip shape, and its effect on stress distributions in the substrate, upon switching from noninvasive to invasive growth, with and without a mechanostat to ensure tip sharpness.

To evaluate whether a soft three-dimensional network can fortify a stiff quasi–two-dimensional shell, we constructed a mechanical finite-element model of this scenario (Fig. 5A, full details in Materials and Methods). Our model consists of a spherical indenter (radius = 5 μm), representing the hyphal tip, composed of a thin cell wall [thickness = 50 nm, *E* = 20 MPa (*26*)] that is filled with an elastic solid, representing the actin structure in the apex, with variable stiffness. The entire sphere is under 1 MPa turgor, as determined experimentally (*8*). The outward turgor places the cell wall in a state of prestress, leading to lateral tensile stresses in the wall of approximately 50 MPa. The indenter is pushed into a soft substrate with *E* = 0.6 MPa, matching the stiffness of the PDMS surfaces used in our experiments.

In the absence of an actin network (*E*_actin_ = 0), indentation at a force of 1 μN, which corresponds to the typical forces applied by *P. infestans* (*8*), results in substantial flattening of the hyphal tip (line in Fig. 5B). Because a part of the mechanical energy is now used to deform the pathogen, the efficiency of stress transfer into the substrate is moderate (Fig. 5B). By contrast, introduction of an infinitely stiff actin network, as the other mechanical limit, completely counters the indenter deformation and thereby results in more efficient stress transfer into the substrate (Fig. 5C).

To analyze this in more detail and for realistic actin network stiffnesses (*48*–*52*), we study the deformation of the shell as a function of indentation force for indenters filled with actin networks of varying Young’s modulus. We use the elastic strain energy in the cell wall as the result of the substrate contact as a measure for the extent to which the tip deforms during indentation. In all cases, the strain energy of the wall increases approximately linearly with the applied force (Fig. 5D). As the actin gel increases in stiffness, the strain energy of the shell for a given indentation force decreases, showing that also soft actin networks are effective in preventing excessive tip deformations. We analyze this further by considering the indentation force F^*^ at which the strain energy in the shell reaches a certain threshold value, here taken as 5 pJ. As shown in Fig. 5E, this force increases with increasing stiffness of the actin gels, or vice versa, if the indentation force increases, a stiffer actin gel is required to maintain the tip shape. These in silico results show that despite a strong stiffness contrast, a soft actin gel is very effective in reducing cell wall deformations and that a stiffer actin gel is needed to maintain tip shape as the invasive process progresses and the forces on the tip grow. This demonstrates the mechanical feasibility of our hypothesized tip shape mechanostat.

These simulation results, together with the experimental data from the preceding sections, lead us to the following hypothesis: During noninvasive growth (middle panel, Fig. 5F), no invasive forces act on the hyphal tip or substrate and the actin cytoskeleton remains in its native configuration, resulting in a rounded hyphal tip prescribed by noninvasive tip growth mechanics (*26*, *27*, *57*). Upon switching to invasive growth, turgor is converted to invasive indentation forces at the tip-substrate contact zone. Without a mechanostat, this would result in substantial tip blunting and a diffusive and inefficient generation of stresses in the substrate (left panel, Fig. 5F). By contrast, in the presence of a mechanostat, the onset of mechanical interactions activates the actin remodeling, which leads to hyphal tip sharpening and localized and efficient force transfer to the substrate (right panel, Fig. 5F).

### Tip shape mechanostasis

**Fig. 6.**
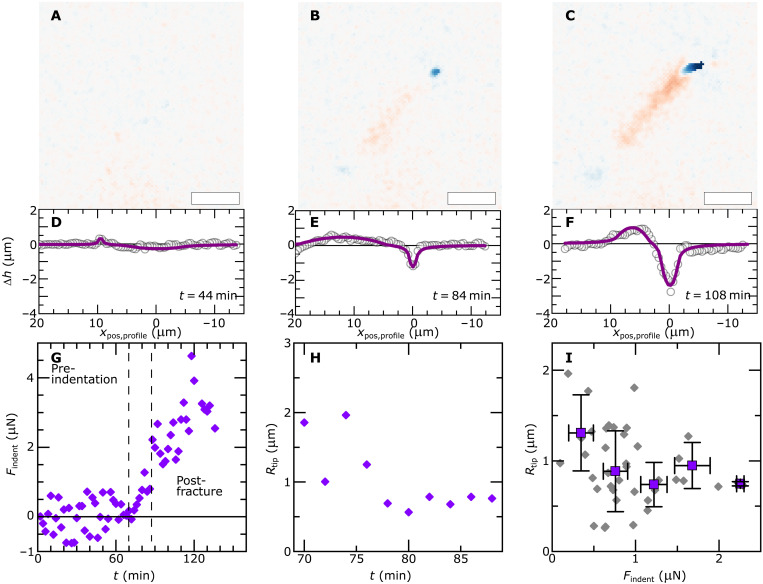
An actin-based tip shape mechanostat. (**A** to **C**) Surface deformation maps during the invasive growth of *P. infestans* Pi-LA-GFP on elastomer surfaces (color code as in Fig. 3, B and D) during the initial noninvasive growth (A), invasive force generation (B), and after surface fracture and substrate penetration (C). (**D** to **F**) Associated surface height profiles (symbols) and fits to a mechanical model for invasive force generation (lines). (**G**) Invasive force applied onto the substrate (*y* axis) as a function of time (*x* axis) for a single cell, showing three regimes. (**H**) In the invasive force generation regime, the tip radius *R*_tip_ (*y* axis) can be extracted from the surface deformation fitting, for the same single cell as in (G). (**I**) Aggregated data for *N* = 5 cells/3 independent experiments, showing the correlation between the tip shape (*y* axis) and the applied invasive force *F*_i_ (*x* axis), revealing initial tip sharpening upon mechanical contact and tip shape stasis at higher forces. Gray diamonds: all data points; purple squares: binned and averaged data, with error bars representing the SD per bin along the force axis. Scale bars, 5 μm (A to C).

To test our hypothesis of actin serving as an adaptive tip shape scaffold, we measured tip shapes and invasive forces during substrate penetration attempts. We again relied on the deformation reporting surfaces developed previously (*8*). In Fig. 6 (A to C), we show a time sequence of surface deformations during the invasive growth of a hypha emerging from a single *P. infestans* cyst, on a soft PDMS substrate. Also, here, we can recognize an initial noninvasive growth phase (Fig. 6A), followed by substrate adherence and invasive force application (Fig. 6B). The force grows over the course of tens of minutes (Fig. 6C) until the surface fractures and the pathogen grows into the elastomer through the crack void. From these two-dimensional surface deformation maps, we can extract a one-dimensional plot of the surface deformation amplitude as a function of position, taken along the contour of the germ tube (Fig. 6, D to F). Using an elastic contact model derived previously (*8*), which treats the invasive hypha as two distinct zones for adhesion and indentation in the limit of linear mechanics, we can fit these deformation profiles with good accuracy up to the moment of penetration (solid lines in Fig. 6, D to F). From these fits, two independent parameters are extracted: (i) the indentation force *F*_indent_, applied by the pathogen at its contact point with the substrate, which governs the depth of the indentation minimum, and (ii) the radius of curvature of the hyphal tip *R*_tip_ at the same position, which determines the width of the indentation minimum as the elastic substrate conforms to the tip at the contact point. For a given cell at a given point in time, we thus obtain both the applied force and the tip shape. The indentation force, shown in Fig. 6G over the course of a host penetration cycle for a single cell, shows the three stages described above. The tip shape analysis is only tractable during the second phase, where the pathogen induces deformations of the surface but has not yet induced a fracture and the behavior obeys linear mechanics. During this phase, we surprisingly observe, for the same cell as shown in Fig. 6G, that the tip radius initially decreases, i.e., the tip sharpens, as the mechanical contact is established, and then reaches a time-independent plateau (Fig. 6H). This induced sharpening of the tip coincides with the formation of the actin aster analyzed in detail above.

Without an adaptive fortification mechanism, the indentation of a surface with a deformable object would invariably lead to tip flattening, i.e., an increase in tip radius, with increasing force (*33*). Here, we observe the exact opposite. We aggregated data for multiple cells (*N* = 6) and plotted the tip radius as a function of the indentation force. This reveals evidence for our hypothesized mechanostat (Fig. 6I). Initially, when the force is zero, the tip has its original shape as dictated by the tip growth mechanism. As the force increases, the actin mechanostat is activated, which coincides with tip sharpening and the subsequent maintenance of a constant and sharp tip shape as the force is increased.

**Fig. 7.**
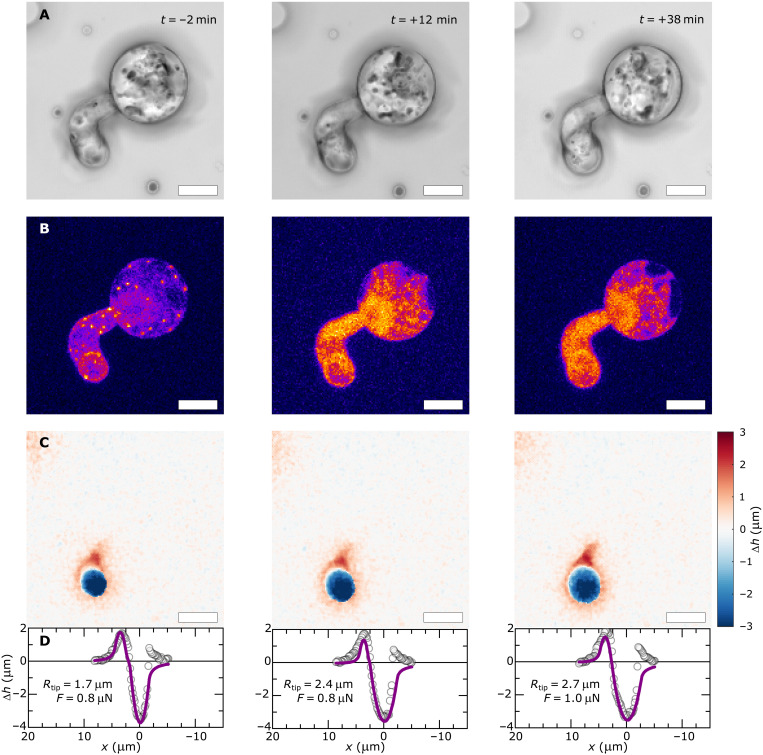
Actin depolymerization inhibits tip-shape mechanostat. Effect of cytoskeletal mechanostat disruption, by acute treatment with 5 mM LatB at *t* = 0, on tip shape and surface mechanics of Pi-LA-GFP during invasive growth. (**A**) Bright-field images before (*t* = −2 min) and after (*t* = +12 and +38 min) LatB treatment. (**B**) Corresponding images from the LifeAct-eGFP channel showing disruption of the actin filaments by the lack of LifeAct-eGFP localization after treatment. (**C**) Corresponding surface deformation maps and (**D**) line profiles of surface deformations from experiments (symbols) and fitted to a mechanical model for invasive growth (solid lines) to extract tip curvature *R*_tip_ and indentation force *F*, as specified in the figure panels. Scale bars in (A) to (C) represent 5 μm; *N* = 6 cells from three independent experiments (see fig. S9 for additional examples).

Our hypothesis involves a causal relationship between the force-induced formation of the actin structure and the ensuing sharpening of the hyphal tip. To evidence this causality, we performed experiments where we disrupted the actin cytoskeleton during invasive force generation by adding a pulse of the drug latrunculin B (LatB), which inhibits actin polymerization and disrupts the actin aster (*31*). We inoculated fluorescent PDMS surfaces with Pi-LA-GFP in water. After 1 hour, when a fraction of the cells is involved in invasive force generation, but has not yet breached the surface, we gently add a drop of a concentrated LatB solution to a final concentration of 5 μM. During this chemical disruption, we simultaneously measured the change in actin distribution and associated alterations of surface deformation patterns. A distinct change in tip shape is visually apparent: The initially sharp tip rapidly blunts in response to the acute LatB treatment (Fig. 7A). This coincides with the dissolution of the actin aster and actin plaques (Fig. 7B) and the distinct broadening of the surface deformation under the hyphal tip (blue zones in Fig. 7C). As before, we fit these surface deformation patterns to our mechanical model to extract the tip shape and indentation force (Fig. 7D), which shows a distinct tip blunting, from the increase in its radius of curvature *R*_tip_. Simultaneously, we observe that the invasive force remains constant, whereas this force was found to grow continuously during unperturbed invasion (*8*). LatB disrupts the actin aster, which results in loss of the mechanostat and thus the blunting of the hyphal tip and the stalling of invasive force generation that prevents building up sufficient force to achieve surface fracture. Additional examples of these effects for *N* = 4 cells from two independent experiments are shown in fig. S9 (A to F). We also recorded data at a lower spatial resolution and higher temporal resolution, shown in fig. S9 (G and H). The tip curvature and force before LatB treatment are indicated by the dashed line: Upon LatB injection at *t* = 0, we observe a rapid tip blunting, by an increase in tip radius (fig. S9G), and an associated reduction in the invasive force (fig. S9H). This experiment reveals that it takes approximately 30 to 40 min upon LatB addition for the tip blunting to reach completion.

We previously showed that these high doses of LatB almost completely suppress the invasivity of *P. infestans*, both on artificial substrates and in potato leaves (*8*). This suggests that the actin mechanostat is crucial for host penetration as it focuses forces to a small area, thereby increasing the invasive pressure at the intended site of entry, and its inhibition leads to not only tip blunting and redistribution of the invasive force over a large area but also the simultaneous stalling of force generation and inhibition of host entry.

## DISCUSSION

We have demonstrated that mechanical stimulation induces a remodeling of the actin cytoskeleton in the filamentous plant pathogen *P. infestans*. Mechanical force induces a transition from a finely meshed network to an aster-like structure composed of thick cables emanating from the locus of mechanical stimulation. This mechano-adaptive response was found to be local, rapid, and quantitative. This mechanically induced remodeling coincides with the sudden sharpening of the hyphal tip at the first stimulus and the maintenance of tip sharpness as the force on the organism grows during host entry. We posed that this constitutes an actin-based mechanostat that ensures hyphal tip sharpness by accommodating increasing mechanical forces on the tip during invasion by adapting the stiffness of the actin network as a mechanical scaffold. A mechanical model for this scenario illustrated the feasibility of this hypothesis, which was verified by our experimental studies of the tip shape evolution during invasive growth.

These findings raise the question how this mechanical feedback is organized. We find that the response is highly localized and occurs within 15 s of stimulation but rapidly vanishes when the stimulus is removed. The local and rapid response appears to rule out a key role for biochemical signals in activating the response; this requires chemical diffusion, which is most likely too slow at this length scale and would lead to diffusive spreading of the effect. Also, transcriptional responses are far slower than the kinetics observed here. Rather, it is more likely that this is an intrinsic response of the actin network mediated by mechano-receptive elements already present in or close to the cytoskeleton.

One possible mechanism for this mechanostat is that it is mediated by actin-associated proteins present in the cytoskeleton during the germination phase. A variety of actin-binding proteins and actin cross-linkers, studied mainly in mammalian cells or in vitro, are capable of rendering actin networks mechano-adaptive. These actin-binding proteins include catch bonding cross-linkers such as α-actinin (*58*) or vinculin (*59*) and the actin-branching protein complex ARP2/3 (*60*, *61*). The *P. infestans* genome contains several genes encoding actin-binding proteins that could be implicated in the mechano-adaptive response, including α-actinin (*62*), ARP2, and ARP3 (*37*, *63*). On the basis of their responses to mechanical stress in other organisms and in vitro studies, these proteins could be involved in orchestrating the mechanostat without requiring a biochemical signaling network. In reconstituted actin networks, studied in vitro, it was found that the mechano-sensitive ARP2/3 complex, which creates branch points in actin filaments, results in force-induced network densification and stiffening (*61*). Also, catch bonding cross-linkers, such as α-actinin, whose bonding strength with actin filaments grows under the action of mechanical stress, can result in force-gated accumulation and filament bundling (*64*, *65*). Cross-linking actin with these proteins does not result in aster formation, which implies that a more complex mechanism involving multiple regulators is at play in the mechanostat.

Last, we note that *Phytophthora* spp. secrete lytic enzymes during host entry (*20*, *66*), a strategy also used by fungi (*14*). Especially, enzymes that target pectins in the host cell walls are of interest here: Pectin provides cell walls with elastic compliance, which is a key determinant in their mechanical resilience. It is unknown how the local degradation of plant cell walls influences the mechanics of host penetration by the naifu invasion mechanism and the mechanostat uncovered here. These enzymes do not sort any effect on our artificial substrates, which are held together purely by silicon-oxygen bonds; the observed effects on these model substrates are thus purely mechanical in origin. However, penetration of real hosts can undoubtedly be facilitated by enzymatic action: Local pectin degradation or demethoxylation can reduce the plant wall cohesion and mechanical resilience, which can, in turn, be speculated to lower the invasive pressure required to achieve penetration. Moreover, local secretion of wall-degrading enzymes can lead to a mechanical weak link in the plant epidermis. Such an induced mechanical heterogeneity could alter the distribution of stresses and enhance the concentration of invasive forces at the intended penetration site, in a very similar way as a mechanical defect concentrates stresses during material failure. Very recently, an approach has been introduced to map local changes in cell wall porosity in in vivo imaging (*67*), which could make it tractable to probe these effects in situ during host penetration on biological hosts; an in-depth exploration of these effects remains a topic for future study.

## MATERIALS AND METHODS

### Cell culture

*P. infestans* 88069 wt, LifeAct-eGFP transformant Pi-LA-GFP (*32*), and a transformant expressing cytosolic GFP (Pi-14-3-GFP) (*38*) were maintained at 18°C in the dark on rye sucrose agar (*68*) supplemented with vancomycin (20 μg/ml), ampicillin (100 μg/ml), and amphotericin B (10 μg/ml), and, in addition, for the transformants with geneticin (5 μg/ml). Fresh zoospores were generated by immersing mature mycelium (9 to 11 days old) with 5 ml of sterilized tap water and incubation for 3 hours in the dark at 4°C during which zoospores are released in the water. The obtained zoospore-rich suspension was decanted from the mycelial plate into plastic tubes and used for experiments, as described in detail in (*8*) and below.

For inoculation of tomato cells, 2 ml of the zoospore suspension (5 × 10^6^ zoospores per ml) was added to 2 ml of a 5-day-old tomato MsK8 cell suspension culture (*40*). For imaging, 30 μl of this mixture was enclosed in a bio-foil slide and incubated overnight at room temperature in the dark. Inoculation of potato plantlets (*69*) was done by applying one droplet (10 μl) containing 5 × 10^4^ zoospores to etiolated stems. After incubation overnight in the dark at room temperature, the plantlets were transferred to a glass bottom imaging dish (MatTek, Ashland, USA) and covered with an agar pad.

For invasion studies on artificial substrates, zoospores were encysted (manual shaking, 1 min) and diluted to a concentration of 10^5^ spores per milliliter, and applied to an 18 mm × 18 mm glass slide, with or without a PDMS coating, in a 80-μl droplet. The slides were mounted in a bespoke three-dimensional printed sample chamber to retain high moisture levels. The digital design file for the three-dimensional printed sample chambers is available from the code repository for this paper. For the cytoskeletal disruption, we inoculated fluorescent PDMS surfaces with an 80-μl zoospore suspension, allowed the cells to incubate for 1 hour, and then added 20 μl of a 25 μM fresh stock solution of LatB to a final concentration of 5 μM.

For phalloidin staining of invasive cells, wt zoospores were encysted as described above and grown on PDMS for 3 hours during which the cysts germinate, and the hyphae invade the substrate. Cells were prefixed using 800 μM m-maleidobenzoyl N-hydroxysuccinimide ester and subsequently fixed by 30-min exposure to a freshly prepared 2% formaldehyde solution followed by transfer to a solution containing 4% formaldehyde, 0.5% glutaraldehyde, 1 mM MgCl_2_, 1 mM CaCl_2_, and 75 mM KCl. Samples were washed in Pipes buffer (2×15 min), followed by staining with phalloidin-rhodamine at 1 μM for 1 hour, washing with buffer (2 × 15 min), and imaging.

### Surfaces

Displacement sensors were produced and characterized as described in (*8*). In brief, a 1:30 Sylgard 184 cross-linker:base mix with fluorophore (15 μl of Pyrromethane 650 at 400 μg/ml per gram of Sylgard) is prepared and degassed (10 min, 1000*g* centrifugation) followed by spin-coating 150 μl on a 18 mm × 18 mm #1 glass slide. Before spin-coating, slides are cleaned with isopropyl alcohol and MilliQ water and dried in an oven at 60°C and exposed to N_2_/O_2_ plasma for 1 min. The spin-coating is a two-stage process: 20 s at 500 rpm for surface wetting, followed by 120 s at 2000 rpm to reduce thickness to a layer of ≈33 μm. After coating with PDMS, the slides are placed in a vacuum chamber for 30 min to remove air bubbles followed by curing at 70°C overnight. The PDMS elastomer is highly optically transparent. Nonetheless, because of the mismatch in refractive index with both the glass onto which it is deposited and the aqueous medium in which our experiments are performed, light refraction at the PDMS-water and PDMS-glass interfaces leads to a reduction in detected fluorescence emission of 16.5% as compared to uncoated glass coverslips (fig. S10). To correct for this, all quantitative metrics used in this study are normalized with respect to the baseline intensity in a posterior section of the germ tube, away from the hyphal tip, and thus report on normalized relative variations within an image such that these results are insensitive to the absolute intensities.

### Imaging

For experiments on plant cell invasion and laser ablation, we used a Roper Spinning Disk Confocal System (Evry, France) consisting of a CSU-X1 spinning disk head (Yokogawa, Japan) mounted on a Nikon Eclipse Ti microscope (Tokyo, Japan) equipped with Perfect Focus system and a 100× Plan-Apochromat 1.4 numerical aperture (NA) oil immersion objective was used for imaging. Fluorescence imaging was performed using a 491- or 543-nm laser line combined with band-pass or long-pass emission filtering (530/50 or 560 nm, Chroma Technology). Images were acquired with a Prime 95B Scientific CMOS camera (Photometrics), controlled by MetaMorph software (Molecular Devices, California). For experiments on PDMS surfaces, images were recorded on a Nikon C2 scanning head mounted to a Nikon Eclipse Ti microscope and equipped with a 60× oil immersion objective (NA = 1.4). For time series measurements, we imaged at the largest field-of-view size, using a 512 pixel × 512 pixel wide view of a region of interest 213 μm × 213 μm in size. Three-dimensional image stacks were acquired once per 2 min, taking 1 min per stack, followed by 1 min without illumination. Each three-dimensional image stack consisted of approximately 41 slices and a z-step size of 0.5 μm. Higher-resolution images were obtained 2 and 4 hours after applying cells to the surface with a field of view of 50 μm × 50 μm at 512 pixels × 512 pixels. All raw data were converted to Tag Image File Format (tiff) without compression before data analysis.

### Laser ablation

For laser ablation, we made use of a high-power pulsed Teem Photonics SNG-03E 532-nm laser (Meylan, France) with a 1000-ns pulse length, which was fed into the Roper Spinning Disk microscope using an iLas 2 FRAP/PA illumination setup (Roper Scientific, Evry, France). Microscope slides containing germinating sporangia were prepared as described in (*32*).

### Data analysis

For laser ablation experiments, images were postprocessed in FIJI (https://imagej.net/Fiji; background subtraction, rolling ball radius 50.0 pixels and linear contrast stretching). For all other experiments, images were analyzed using custom-built scripts in Matlab 2018b. In short, all images were imported after which a region of interest containing a single cell was selected. For surface deformation profiling, we refer to a detailed description in (*8*). Actin accumulation in the hyphal tip, expressed by the scalar α, was determined from the XZ projections of three-dimensional image stacks, recorded by imaging LifeAct-eGFP, using a home-written algorithm. This procedure first traces the centerline of the hyphae from cyst to tip, locally averaging the LifeAct-eGFP intensity over a small window to segment the hyphae. Then, it computes the intensity profile along this tube contour to compute the actin accumulation. We have verified that the orientation of the projection has no qualitative effect on the results (see the Supplementary Materials).

### Fitting surface deformations

We use a mechanical model to analyze the indenter geometry during the invasion process by fitting experimentally obtained surface deformation profiles. The mechanical model, described in detail previously (*8*), relies on balancing indentation and adhesion forces, applied by ellipsoidal elements that are spatially separated but mechanically coupled and that are both modeled as ellipsoidal Hertzian contact sites. We assume a Poisson ratio of 0.45 for the PDMS, and a modulus of 0.58 MPa as was previously measured (*8*). The model assumes an invasion angle of 49°, also determined from experiments (*8*), and fits five geometrical parameters to a displacement profile of the surface in an iterative process. The geometrical parameters are the width/height of both indentation and adhesion sites and the distance between these sites. In this study, we use this procedure to extract the radius of curvature of the indentation site at the hyphal tip, defined as the smallest dimension of the indenter.

### Finite-element simulations

To study the mechanical deformations in both the tip and the substrate during tip invasion, we develop a finite element model. We model the tip as a hemispherical shell of radius *R* = 5 μm that is supported by an elastic actin gel at the interior. The cell wall of the tip is treated as a thin elastic shell of thickness *h* = 50 nm, Young’s modulus *E*_wall_ = 20 MPa, and Poisson ratio *v*_wall_ = 0.3 (*26*, *70*, *71*). While the mature cell wall has a modulus on the order of several gigapascals (*70*, *71*), the lower value used here reflects the cell wall softening that occurs to allow for tip growth (*26*). The internal turgor pressure *P* in the tip results in a prestress in the cell wall, which can be estimated as σ_θθ,0_ = σ_φφ,0_ = *PR*_0_/(2*h*), where *R*_0_ is the radius of the stress-free tip (*72*). In our calculations, we assume *P* = 1 MPa, as determined previously for *Phytophthora* (*8*), leading to lateral stresses in the cell wall in the order of 50 MPa. The cell wall is supported by an actin gel, which we consider as an isotropic and linearly elastic material that fills the complete tip. The gel is characterized by a Young’s modulus *E*_gel_, which we vary in our calculations, and a Poisson ratio *v*_gel_ = 0.4 (*73*). Last, the substrate is treated as a linearly elastic isotropic solid with Young’s modulus *E*_sub_ = 0.6 MPa and Poisson ratio *v*_sub_ = 0.45 (*8*). The indentation process is modeled by applying a prescribed displacement to the lower boundary of the elastic substrate while keeping the top of the tip fixed. The contact between the tip wall and the substrate is implemented using an augmented Lagrangian formalism, which effectively prevents overlap of the tip and the substrate. The equilibrium shape of the tip and substrate, the local stresses, and the resulting indentation force are found by minimizing the total elastic energy using the finite element method, as implemented in Comsol Multiphysics. We use quadratic elements on a quadrilateral mesh for the elastic substrate and a triangular mesh for the actin gel, which is refined in the contact region. We have checked the accuracy of the solutions by performing calculations for different mesh parameters. The contact problem leads to a highly nonlinear system of equations, which is solved iteratively using Newton’s method. Small incremental steps in the prescribed displacement had to be used for the solution to converge. Convergence issues did not allow us to obtain results for large indentation depths; therefore, we have only considered the initial stages of indentation when the forces and displacements are relatively small.

## Notes

View/request a protocol for this paper from *Bio-protocol*.
