# Supplementary material for: An actin mechanostat ensures hyphal tip sharpness in Phytophthora infestans to achieve host penetration
Source: Sci Adv. 2022 Jun 10;8(23):eabo0875. doi: 10.1126/sciadv.abo0875 (PMC9187236; doi:10.1126/sciadv.abo0875)
Supplement: Supplementary file 1 — Figs. S1 to S10 Table S1 [file sciadv.abo0875_sm.pdf]

Science Advances
AAAS

Supplementary Materials for

An actin mechanostat ensures hyphal tip sharpness in *Phytophthora infestans* to achieve host penetration

Jochem Bronkhorst *et al.*

Corresponding author: Tijs Ketelaar, tijs.ketelaar@wur.nl; Joris Sprakel, joris.sprakel@wur.nl

Sci. Adv. **8**, eabo0875 (2022)
DOI: 10.1126/sciadv.abo0875

The PDF file includes:

Figs. S1 to S10
Table S1
Legends for Movies S1 to S6

Other Supplementary Material for this manuscript includes the following:

Movies S1 to S6

Computational analysis of image data

Image analysis was performed on home-developed scripts written in MatLab; all original code files and extended documentation are available publicly from: https://github.com/jorissprakel/phytophthora_mechanostat

Actin accumulation in hyphal tips:
We used confocal microscopy to obtain three-dimensional image stacks of individual germ tubes during pressure application onto an artificial substrate. For analyzing the F-actin density, visualised by the LifeAct-eGFP reporter In *Phytophthora infestans* transformant Pi-LA-GFP, we first constructed maximum intensity projections either as a top view (xy projection) or side view (xz projection). We have chosen this approach because we found that maximum projections enable comparison of the most fluorescent region independent of hyphal thickness, making comparisons more robust and independent of local cell morphology.

From these projections, we first determined the hyphal centre line (indicated as a yellow line in main text Figure 1I) using an iterative skeletonization function. We note that the determined centre line never extends fully into the tip edge due to the reduction algorithm, which can lead to an underestimation of the accumulation factor α in extremely polarized cells, although rarely observed. We then recorded the fluorescence intensity along this centre line, averaged for each point on the centre line over the entire width of the germ tube. From the trace of fluorescence intensity versus position in the germ tube obtained in this way we computed the accumulation factor $\alpha = \frac{I_{max}}{I_{base}} - 1$, where I_{max} is the peak intensity, located at or near the tip, and I_{base} the mean baseline intensity, recorded in the posterior section of the germ tube. If the F-actin concentration, judged from the LifeAct intensity, is homogeneous along the germ tube, $\alpha = 0$, while strong tip-accumulation gives $\alpha >> 0$.

Additional examples of this analysis are shown in Figure S4. We have confirmed that both, side (xz) and top (xy) projections give results that are qualitatively similar as shown in Figure S5.

Displacement analysis:
Our approach to measure the pathogen induced surface deformations, expressed as the displacement Δh along the surface normal, was described in detail before in Bronkhorst et al. Nature Microbiology 6, pp. 1000-1006, 2021. In short: we record three-dimensional image stacks of a fluorescent PDMS in contact with a non-fluorescent aqueous medium. To extract, for each xy-pixel, the surface location in z, denoted here as Δh, where Δh = 0 is the position of the substrate in area without deformation, we fit the intensity decay at the PDMS-medium interface to a sigmoidal function. We then perform a tilt correction, to resolve small tilt angles of the surface with respect to the imaging plane. This results in spatial (xy) maps of surface displacements (Δh), which are used in subsequent analysis.

Deformation-actin correlation analysis

We performed analyses to correlate, both spatially and temporally, the local surface displacement (Δh) with the local intensity of LifeAct-eGFP, as a F-actin marker. The code computes for each pixel the relative LifeAct-eGFP fluorescence and links this to the local displacement from the analysis described above. We then divided the data in 3 zones; adhesion zones, where $\Delta h > +0.5\mu m$ (approximately 5x the surface displacement resolution), zones of no to weak mechanical interactions, where $+0.5\mu m > \Delta h > -0.5\mu m$, and indentation zones, where $\Delta h < -0.5\mu m$ (approximately 5x the surface displacement resolution), and aggregated fluorescence intensity data for these different zones.

Additional data description

Cytoplasmic GFP reporter

To verify that the observed increase in actin accumulation during pressure application, and prior to substrate entry, is not the result of accumulation of cytoplasm containing unbound LifeAct reporter, we performed control experiments on a *P. infestans* transformant that expresses a cytoplasmic GFP (Pi-14-3-GFP). We performed three-dimensional confocal imaging both at higher frame rates and with lower resolution (**Error! Reference source not found.**A-B) and at lower frame rates with higher resolution (**Error! Reference source not found.**C-D). Upon contact and at the start of invasion a small increase in fluorescence is at times observed; this feature is substantially weaker as compared to the LifeAct actin reporter line Pi-LA-GFP, and does not show any microstructural features as expected. Only *after* host penetration, when cytoplasmic and nuclear repositioning are expected to occur to initiate the invasion process inside the host, we do observe a substantial accumulation of cytoplasm in the tip. This confirms that our reported results for the accumulation of actin in the hyphal tip prior to, and during penetration are not the result of random cytoplasmic streaming but due to mechanical feedback to the cytoskeleton.

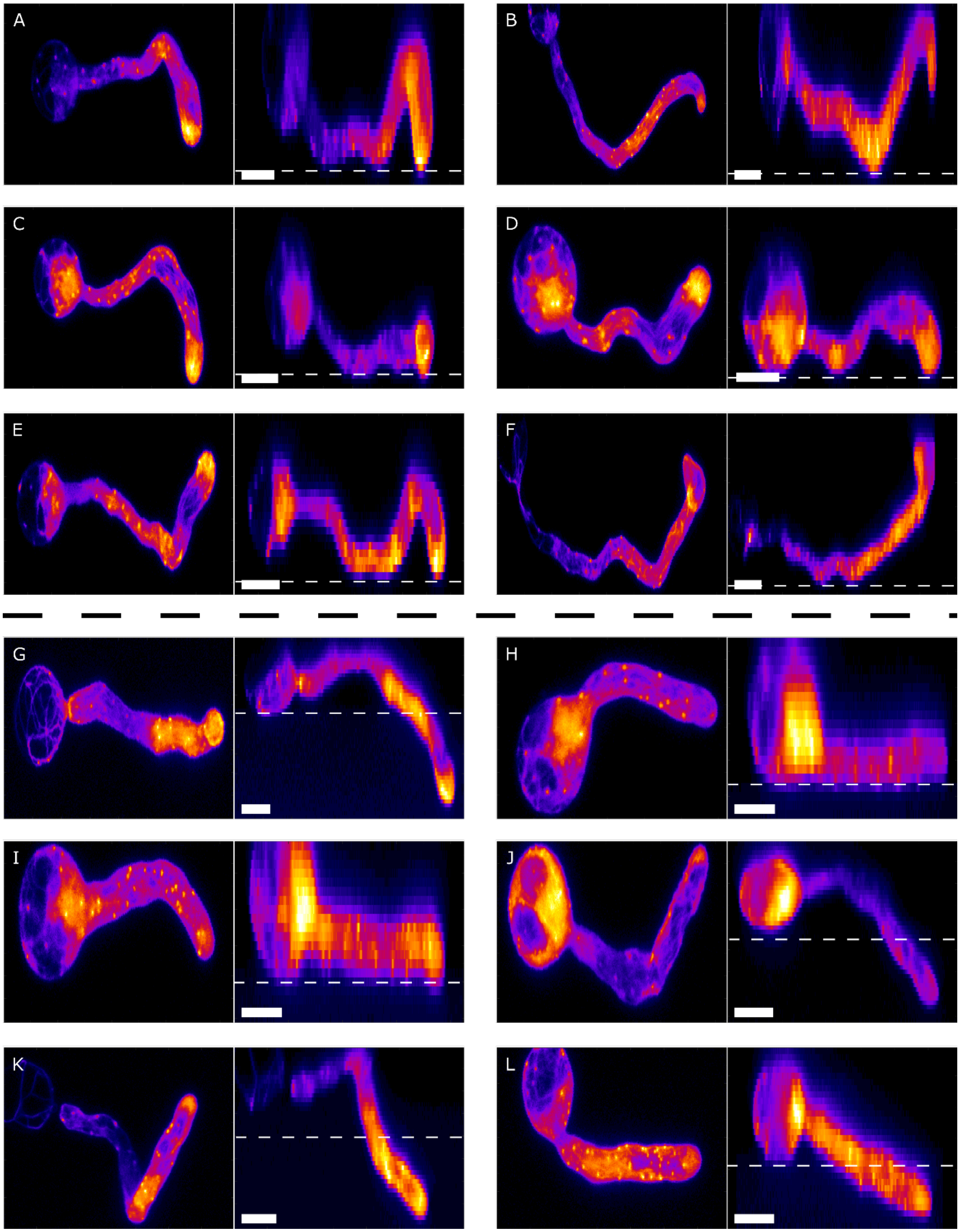

Figure S1 *Additional examples of the actin cytoskeletal structure in Pi-LA-GFP during force application and penetration, all taken 4hpi on either glass (A-F) or PDMS (G-L), in a maximum intensity top view projection (xy, left) and side view projection (xz, right). Dashed lines indicate the substrate-medium interface. Scale bars represent 5 μm.*

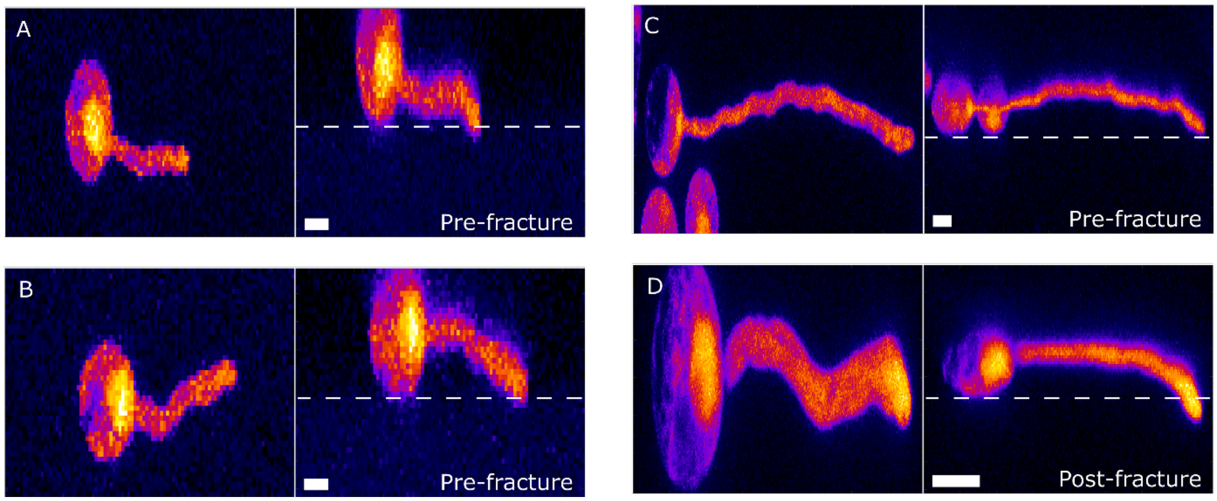

Figure S2 *Confocal images of Pi-14-3-GFP on PDMS surfaces, showing a maximum intensity top (xy, left) and side (xz, right) projection. A-B: lower resolution from dynamic imaging, C-D: higher resolution images with a lower acquisition rate. Scale bar represents 5 μm.*

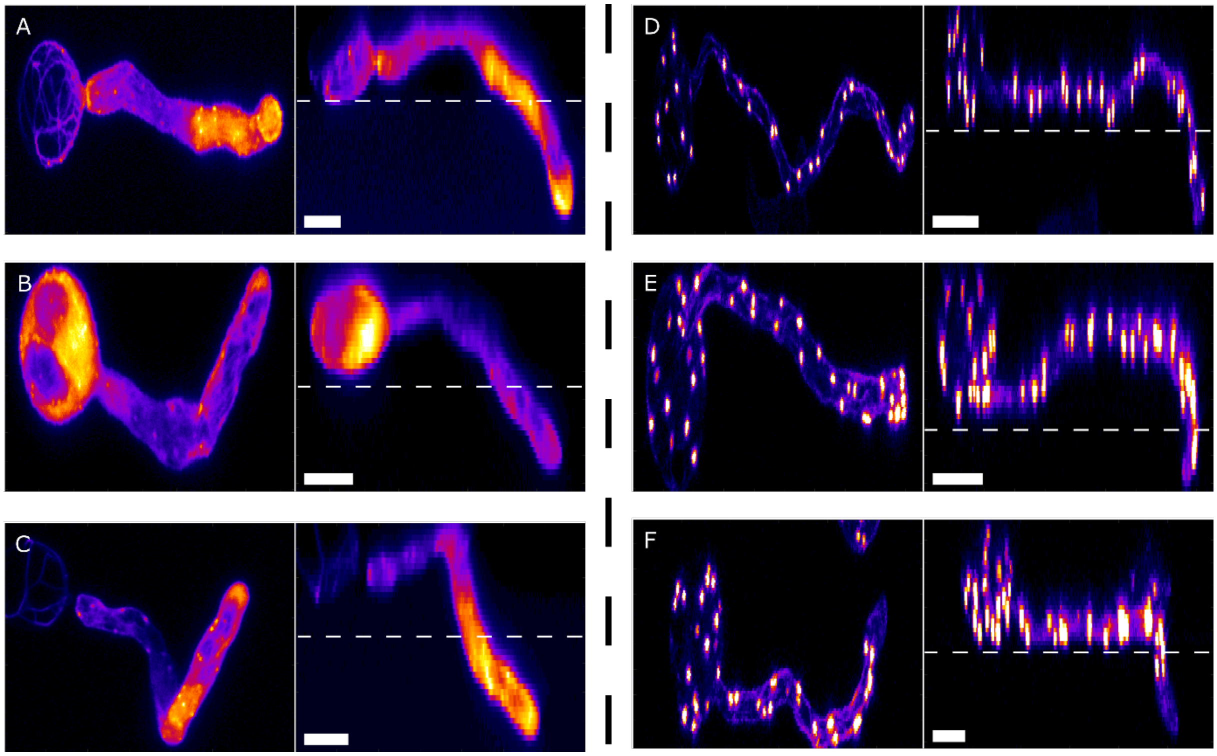

Figure S3 *Comparison of actin cytoskeleton imaging in* Phytophthora infestans *with a genetically-expressed LifeAct-eGFP actin marker (Pi-LA-GFP) (A-C) and a phalloidin-rhodamine conjugate staining of fixed wt-Pi cells (D-F), all recorded during penetration of PDMS substrates. Each image shows maximum intensity top (xy, left) and side projections (xz, right). Scale bar represents 5 μm.*

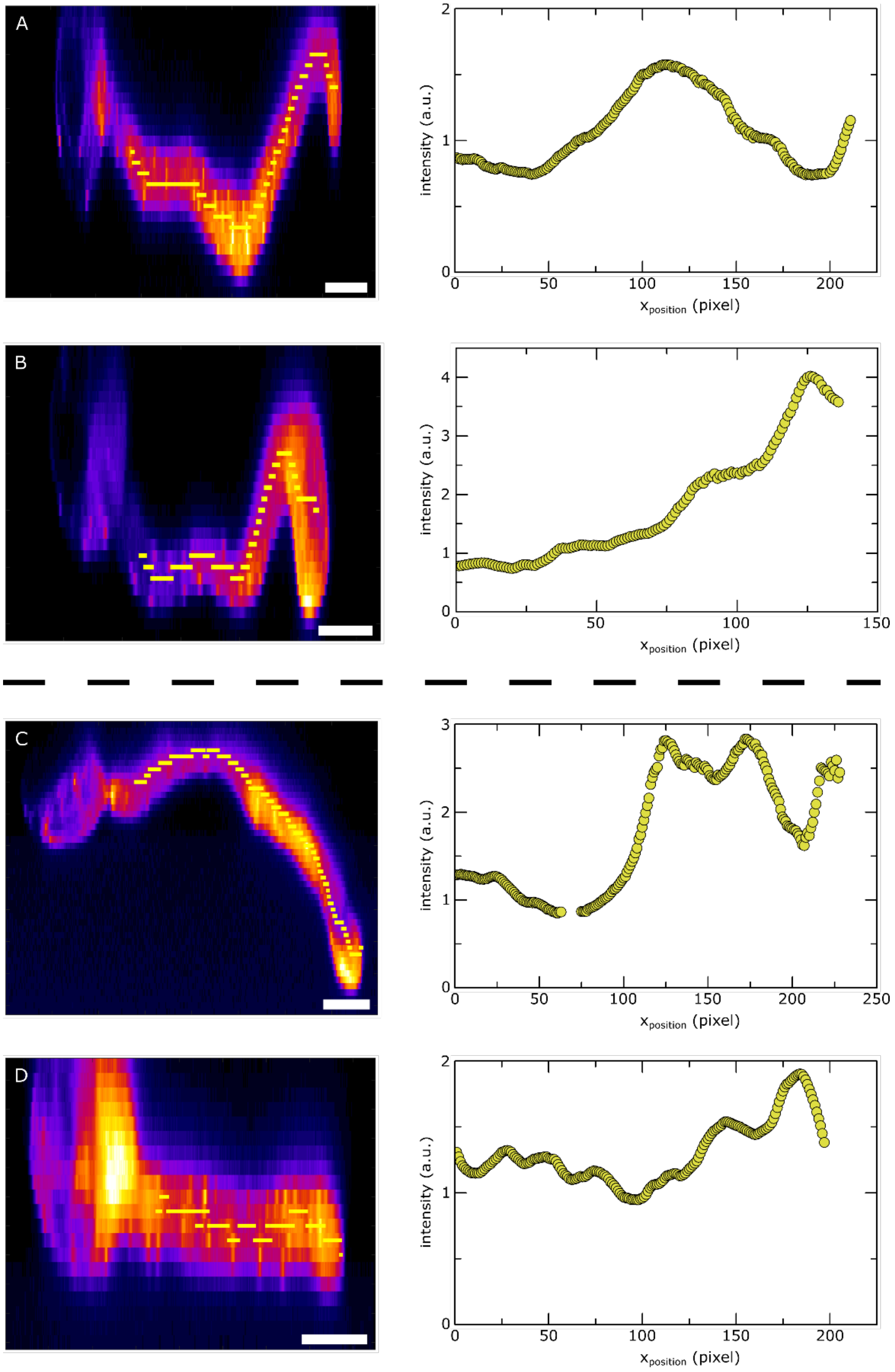

Figure S4 *Additional examples of the centre line detection (left) and LifeAct-eGFP intensity profile (right) in maximum intensity xz projections for Pi-LA-GFP (2hpi) on glass (A-B) and PDMS (C-D). Scale bars represent 5 µm.*

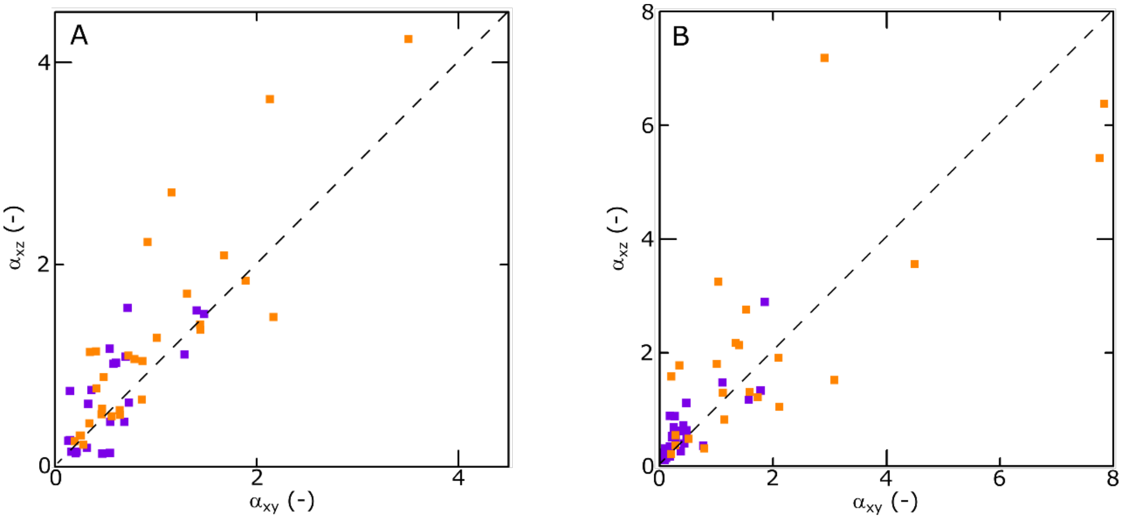

Figure S5 *Comparison of the values of the accumulation parameter α from side view (xz) versus top view (xy) maximum intensity projections, at 2hpi (A) and 4hpi (B). Purple squares represent data on PDMS substrates, orange squares on glass substrates. Dashed line is a guide to the eye that indicates perfect correlation.*

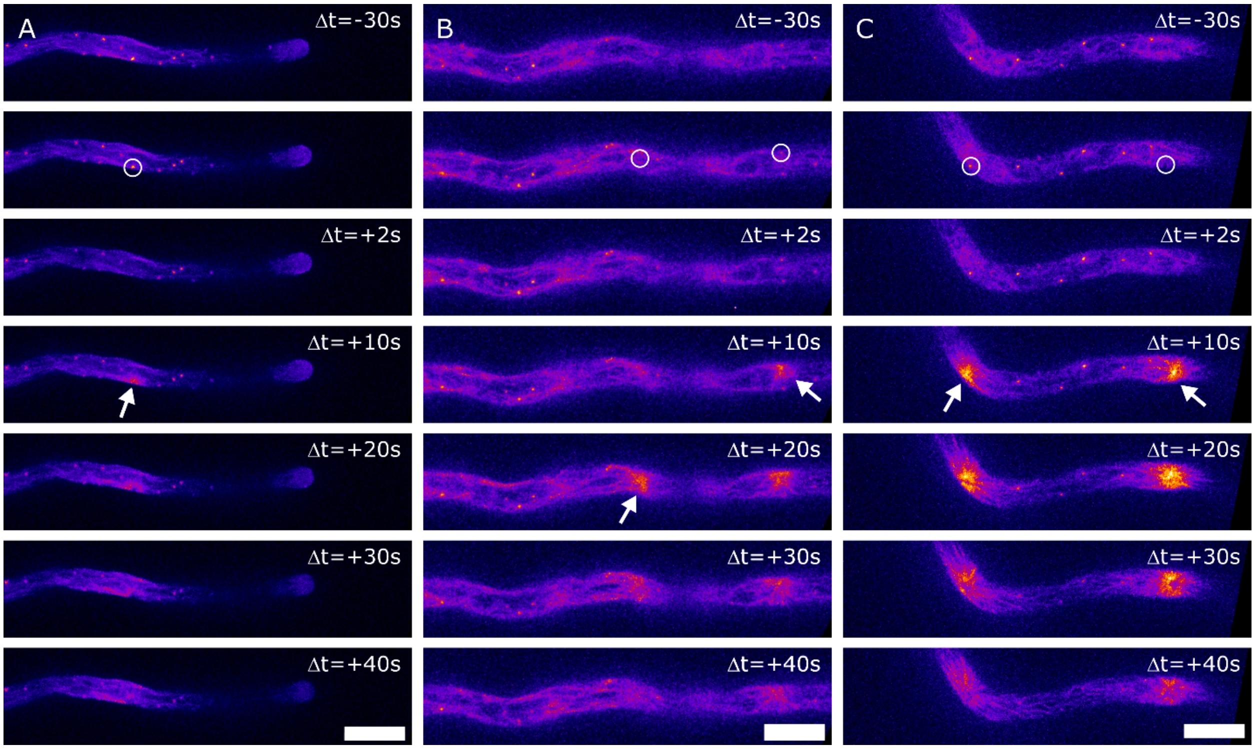

Figure S6 *Additional data from laser ablation experiments on Pi-LA-GFP. Approximately 10-20 seconds post ablation a fluorescent response of the actin network is observed in the form of an aster-like configuration (arrow indicates first appearance). Scale bar represents 5 μm. The white circles indicate target locations of the ablation laser.*

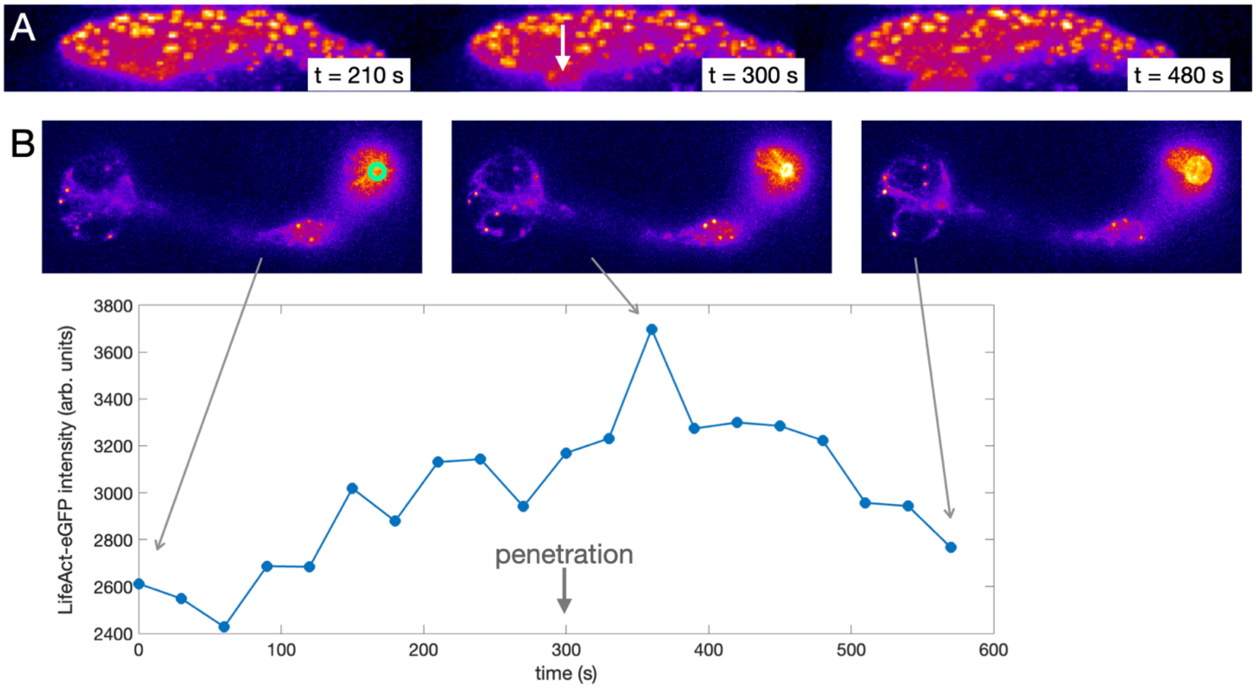

Figure S7 *Additional analysis on a unique observation (N=1) of Pi-LA-GFP invasion of a potato plantlet, belonging to the data shown in main text Figure 2C&E. a) Side view (xz maximum intensity projection) to identify the time of penetration by the formation of a protrusion of the pathogen into the host tissue, occurring at the time point 270-300s in this time sequence. b) Analysis of the local LifeAct-eGFP intensity (in the area indicated by the green circle) at the hyphal apex, showing a transient increase in LifeAct-eGFP intensity, signaling the formation of the actin aster, whose intensity peaks 1 minute after penetration, after which the signal gradually decreases over the course of several minutes.*

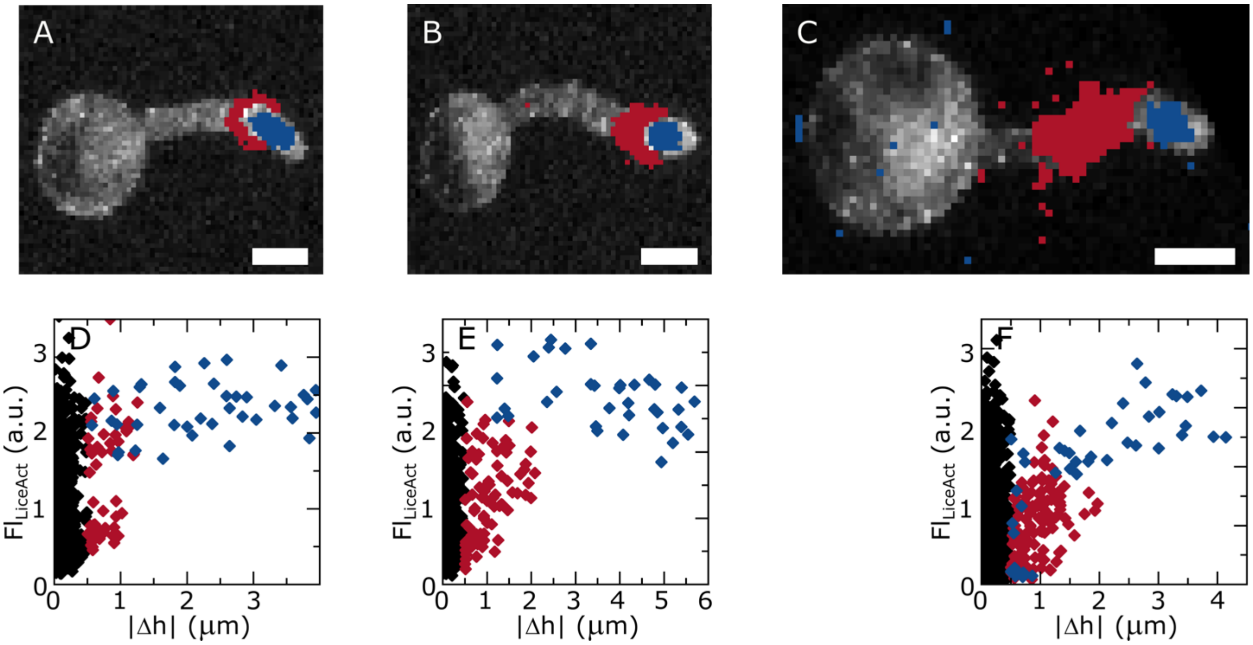

Figure S8 *Additional examples of the data shown in main text Figures 4E-F. Top row A-C: overlay of identified adhesion (red) and indentation sites (blue) on the xy-projection of the LifeAct eGFP signal for a single cell showing strong localization in two distinct zones. Bottom row D-F: corresponding scatterplots, showing the local LifeAct eGFP intensity (Y-axis) and absolute surface deformation amplitude for each pixel at a single time point for a single cell (X-axis); data is grouped into adhesive (red), indentation (blue) and non-interacting sites (black) on the basis of the sign and amplitude of the local surface deformation (as explained in main text).*

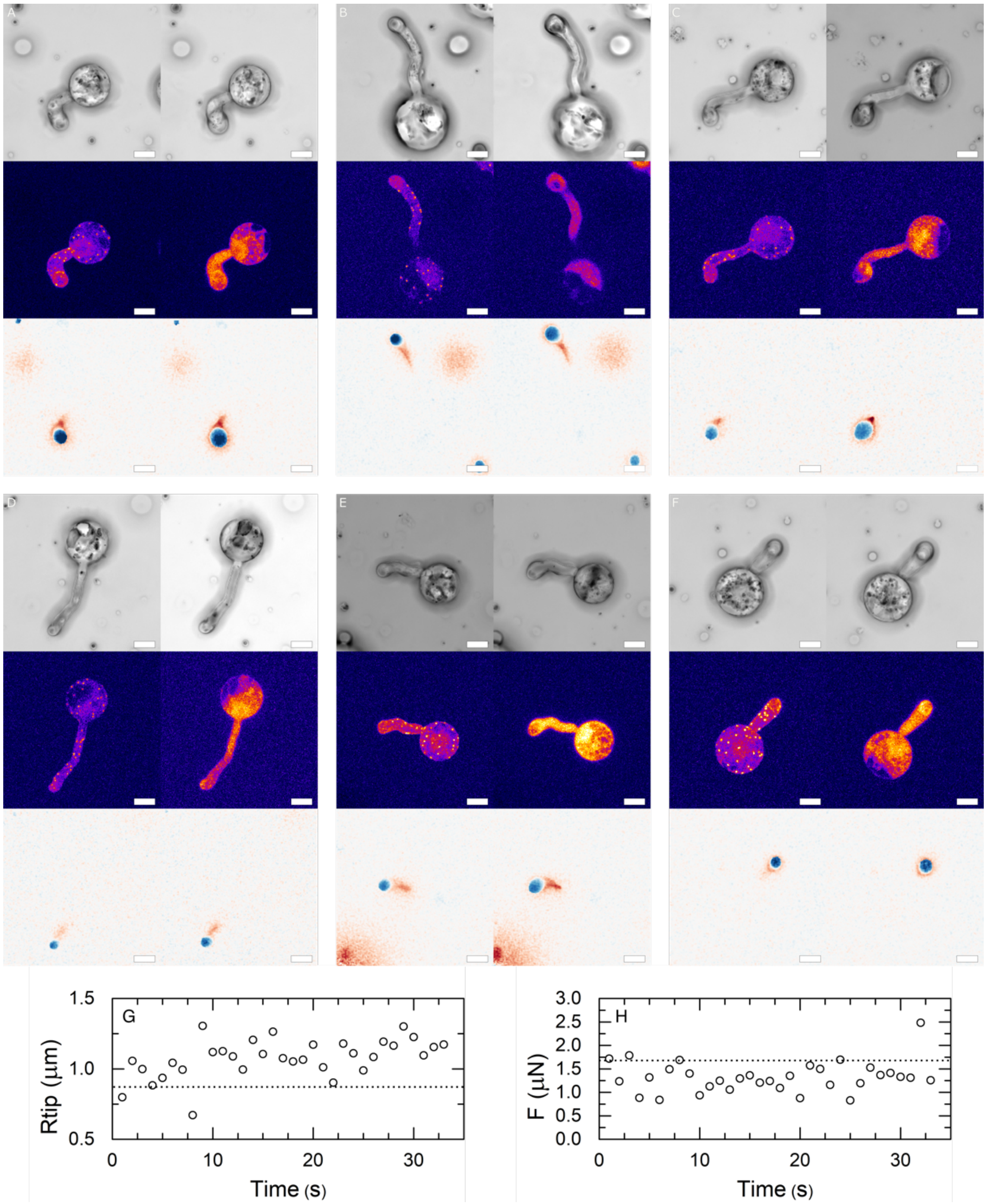

Figure S9 A-F: *Additional examples (N=6 cells from 3 independent experiments) that visibly show tip widening upon LatB treatment. For each cell in A-D the same cell is shown immediately prior to LatB treatment (right, t=-2 min), and after LatB addition (left, A=38min, B=43min, C=38min, D=38min, E=76min, F=37min). Top rows show the brightfield channel, middle row the LifeAct-eGFP channel and bottom row the surface deformation map, in which the tip blunting is visible in the expansion of the indentation zone (blue). Scale bars represent 5 μm. G-H: kinetics of tip reshaping upon LatB treatment (at t=0) for a cell recorded at a lower spatial but higher temporal resolution showing the transient change in tip curvature (G) and indentation force (H). Dotted line indicates the tip curvature (G) and force (H) just prior to LatB injection.*

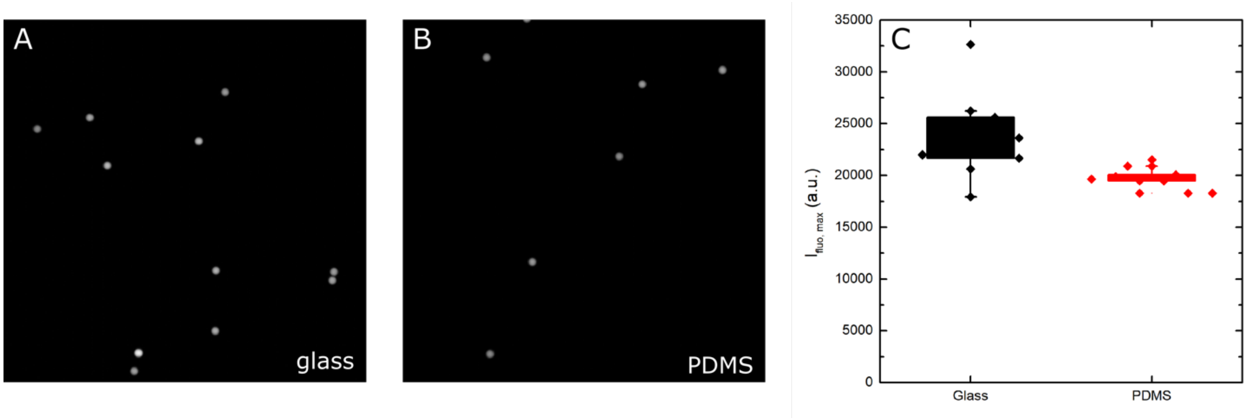

Figure S10 *Quantification of the reduction in fluorescence emission detection in confocal microscopy experiments as the result of the PDMS elastomer layer on glass slides: A-B) confocal microscopy images of fluorescein-labelled melanin microparticles (nominal diameter = 5 mm), acquired using identical excitation/emission settings as during GFP-marker line imaging on both uncoated glass (A) and PDMS-coated glass (B). C) Peak intensity comparison shows a modest 16.5% reduction in fluorescence transmission to the confocal detector. N=10 beads per substrate.*

Table S1. Summary of experimental statistics

Experiment	Figure	Sample numbers
**Actin structure**	Figure 1 A-B, E-F<br>Figure 1 C-D, G-H<br>Figure 1 K<br>Figure 1 L	0.56 MPa PDMS Pi-LA-GFP<br>• N = 32 (from 3 independent experiments)<br>Glass Pi-LA-GFP<br>• N = 45 (from 3 independent experiments)<br>0.56 MPa PDMS Pi-LA-GFP<br>• N = 10 (from 6 independent experiments)<br>0.56 MPa PDMS Pi-LA-GFP<br>• 2hpi: N = 31 (from 4 independent experiments)<br>• 4hpi: N = 27 (from 2 independent experiments)<br>Glass Pi-LA-GFP<br>• 2hpi: N = 32 (from 3 independent experiments)<br>• 4hpi: N = 27 (from 3 independent experiments)
**Cell / stem penetration**	Figure 2 A-B (MsK8 tomato cells)<br>Figure 2 C-E (Etiolated potato stem section)	Pi-LA-GFP<br>• N = 4 (from 1 independent experiment)<br>• N = 1 (from 1 independent experiments)
**Laser ablation**	Figure 3	Pi-14:3-GFP (Control)<br>• N = 5 (from 1 independent experiment)<br>Pi-LA-GFP<br>• N = 10 (from 2 independent experiment)<br>• Plaques: N = 10 (from 1 independent experiment)<br>• Non-plaques: N = 9 (from 1 independent experiment)<br>• Distal: N = 10 (from 1 independent experiment)<br>• Tip: N = 9 (from 1 independent experiment)
**Deformation & LifeAct-GFP correlation**	Figure 4 A-F	0.56 MPa PDMS Pi-LA-GFP<br>• N = 6 (from 5 independent experiments)
	Figure 4 H	0.56 MPa PDMS Pi-LA-GFP<br>• N = 6 (from 5 independent experiments)
**Force and tip curvature fitting**	Figure 6	0.56 MPa PDMS Pi-LA-GFP<br>• N = 5 (from 3 independent experiments)
**LatB disruption**	Figure 7	0.56 MPa PDMS Pi-LA-GFP + 5 μM LatB<br>• N = 6 (from 3 independent experiments)

Captions for supplementary movies

Supplementary Video 1: Movie_S1.mp4
LifeAct-GFP expressing *P. infestans* strain (left) and displacement map (right) of a non-invasive hypha: hypha is growing from original cyst body on the PDMS surface, with no clear displacement seen and uniform distribution of GFP signal. Time between each frame is 2 minutes, cyst size is approximately 15 µm, hypha approximately 2-3 µm, displacement colorbar is in µm units.

Supplementary Video 2: Movie_S2.mp4
LifeAct-GFP expressing *P. infestans* strain (left) and displacement map (right) of a invasive hypha: hypha is growing from original cyst body on the PDMS surface, with clear deformation of the substrate and a localised increase of LifeAct-eGFP signal at the contact point. Time between each frame is 2 minutes, cyst size is approximately 15 µm, hypha approximately 2-3 µm, displacement colorbar is in µm units.

Supplementary Video 3: Movie_S3.mp4
Cytoplasmic GFP expressing *P. infestans* strain in XY projection (left) and XZ projection (right) of an invasive hypha: hypha is growing from original cyst body on the PDMS surface, without a large increase in fluorescence during invasion. Time between each frame is 2 minutes, cyst size is approximately 15 µm, hypha approximately 2-3 µm.

Supplementary Video 4: Movie_S4.mp4
Displacement map of an invading *P. infestans* hypha (left) and profile along green '+' markers (right): the right plot is the displacement profile (blue circles), which are fitted with the Naifu invasion model (red line). The model output contains the geometrical shape of the indentor and the applied force, which are used in the study. Time between each frame is 2 minutes.

Supplementary Video 5: Movie_S5.mp4
Invasive LifeAct-GFP construct expressing *P. infestans* strain in XY projection: movie is a flyaround of an invasive hypha, scale bar is 10 µm. Note that the original cyst is resting on the PDMS (dyed slab was measured, channel not shown), so the cysts has penetrated the PDMS substrate by several micrometers.

Supplementary Video 6: Movie_S6.mp4
Non-invasive LifeAct-GFP expressing *P. infestans* strain in XY projection: movie is a flyaround of a non-invasively growing hypha, scale bar is 10 µm. Note that the original cyst is resting on the PDMS (dyed slab was measured, channel not shown), and that intensity along the germ tube is relatively uniform.
